# A Bayesian mixture model for the analysis of allelic expression in single cells

**DOI:** 10.1038/s41467-019-13099-0

**Published:** 2019-11-15

**Authors:** Kwangbom Choi, Narayanan Raghupathy, Gary A. Churchill

**Affiliations:** The Jackson Laboratory, 600 Main Street, Bar Harbor, ME 04609 USA

**Keywords:** Bioinformatics, Gene expression analysis, Computational models, Statistical methods

## Abstract

Allele-specific expression (ASE) at single-cell resolution is a critical tool for understanding the stochastic and dynamic features of gene expression. However, low read coverage and high biological variability present challenges for analyzing ASE. We demonstrate that discarding multi-mapping reads leads to higher variability in estimates of allelic proportions, an increased frequency of sampling zeros, and can lead to spurious findings of dynamic and monoallelic gene expression. Here, we report a method for ASE analysis from single-cell RNA-Seq data that accurately classifies allelic expression states and improves estimation of allelic proportions by pooling information across cells. We further demonstrate that combining information across cells using a hierarchical mixture model reduces sampling variability without sacrificing cell-to-cell heterogeneity. We applied our approach to re-evaluate the statistical independence of allelic bursting and track changes in the allele-specific expression patterns of cells sampled over a developmental time course.

## Abstract (web summary)

Allele-specific expression at single-cell resolution can reveal stochastic and dynamic features of gene expression in greater detail. The authors propose scBASE, a soft zero-and-one inflated model that improves estimation of cellular allelic proportions by pooling information across cells.

## Introduction

Allelic imbalance of transcripts is common across many genes^1^. It can range from a subtle imbalance to complete monoallelic expression as in imprinted genes^2^ or genes under dosage compensation by X chromosome inactivation^3,4^. Single-cell RNA sequencing (scRNA-Seq) can reveal features of cellular gene expression that cannot be observed in bulk RNA sequencing^5^. In single cells, allelic proportions often form U-shaped or W-shaped distributions due to the occurrence of monoallelic transcriptional bursts^6^. However, our ability to discern gene expression dynamics is limited by low depth of sequencing coverage per cell^7–14^ and thus it is critical to make full use of all information available in scRNA-Seq data.

**Fig. 1:**
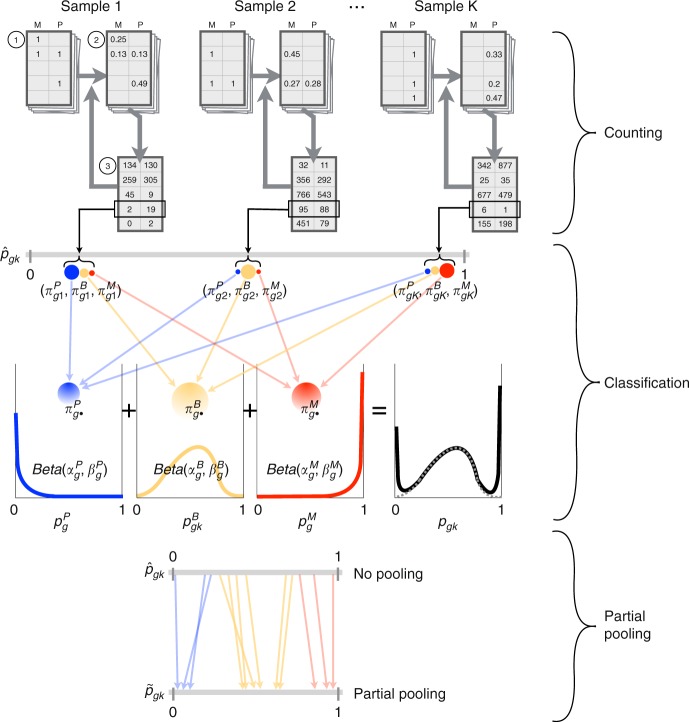
Overview of the ${\mathtt{scBASE}}$ algorithm. The Counting step estimates the expected read counts using an EM algorithm to compute a weighted allocation of multi-reads. Each read is represented as an incidence matrix that summarizes all alignments to genes and alleles ①. Weighted allocation of multi-reads uses a current estimate of allele-specific gene expression to compute weights equal to the probability of each possible alignment ②. The weights are summed across reads to obtain the expected read counts for each gene and allele ③. Steps ② and ③ are repeated until the read counts converge. The weighted allocation estimates of maternal allelic proportion (${\hat{p}}_{gk}$) are obtained at this step. The Classification step computes the posterior probability of paternal monoallelic (P), bi-allelic (B), or maternal monoallelic (M) expression (${\pi }_{gk}^{s}$) using current estimates of the model parameters (Equation 3 in Supplementary Methods). The classification model is a beta-binomial mixture model with three components. The model parameters are initialized to non-informative values and are obtained from the partial pooling step in subsequent iterations. The partial pooling step uses the classification results to re-estimate the weights of mixture components (${\pi }_{g\cdot }^{s}$) and parameters of the Beta densities (${\alpha }_{g}^{s},{\beta }_{g}^{s}$) that define the distribution of the within-class the maternal allelic proportions (${p}_{g}^{s}$). The partial pooling estimate of the maternal allelic proportions (${\tilde{p}}_{gk}$) is obtained as an average of the class-specific proportions weighted by the class membership probabilities (Eq. 1 in Methods)

We propose an approach to classification and estimation of allele-specific gene expression in single cells (Fig. 1 and Methods). We first count the allele-specific read alignments using one of two methods. In the unique-reads method, we exclude multi-mapping reads (multi-reads) and count only reads that map unambiguously to one allele of one gene. Alternatively, we can include multi-reads using an expectation-maximization (EM) algorithm to estimate counts by weighted allocation^15–17^. Next, we compute a probabilistic classification of each gene in each cell into paternal monoallelic, bi-allelic, or maternal monoallelic expression states. Lastly, we can apply partial pooling to improve the individual cell-level estimation by combining information across cells that are in the same allelic expression state. The classification and partial pooling steps inform one another and are applied iteratively. Partial pooling can be applied to either of the read counting results leading to four different methods for estimating allelic proportions: (a) unique reads, (b) weighted allocation, (c) unique reads with partial pooling, or (d) weighted allocation with partial pooling. These methods are implemented in our ${\mathtt{scBASE}}$ software.

In the following sections, we examine the effects of weighted allocation and partial pooling on classification and estimation of allelic proportions. We then apply ${\mathtt{scBASE}}$ methods to evaluate the statistical independence of allelic bursting. Finally, we illustrate the interpretive power of allelic expression analysis of scRNA-Seq using data from a development time course^8^.

## Results

### Application of ${\mathtt{scBASE}}$

We applied ${\mathtt{scBASE}}$ methods to scRNA-Seq data from 286 pre-implantation mouse embryo cells from an F1 hybrid mating between female CAST/EiJ (CAST) and male C57BL/6J (B6) mice^8^. Cells were sampled along a time course from the zygote and early 2-cell stages through the late blastocyst stage of development. We created a diploid transcriptome from CAST- and B6-specific sequences of each annotated transcript (Ensembl Release 78)^18^ and aligned reads from each cell to obtain allele-specific alignments. In order to ensure that genes had sufficient polymorphic sites for ASE analysis, we restrict attention to 13,032 genes that had at least four allelic unique reads in at least 10% of cells. Where indicated below, we apply ${\mathtt{scBASE}}$ to only 122 cells from the blastocyst stages of development, or to only 60 cells in the mid-blastocyst stage.

### Discarding multi-reads increases spurious ASE calls

**Fig. 2:**
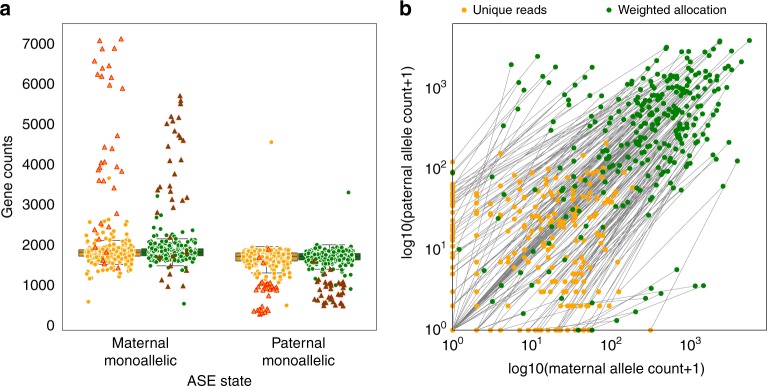
Weighted allocation of multi-reads reduces monoallelic expression calls. **a** For each of 13,032 genes, we obtained the allele-specific read counts by unique reads and by weighted allocation. We counted the numbers of genes in each cell that showed either maternal or paternal monoallelic expression. Each data point in this figure represent a cell. Yellow and green circles indicate unique-reads and weighted allocation respectively, for all 286 cells. The zygote and 2-cell stage cells (highlighted in red triangles) have large numbers of genes with maternal monoallelic expression. On average there are $\sim$66 fewer monoallelic calls per cell with the weighted allocation counts. The outlier cell with high levels of paternal monoallelic expression was noted in Deng et al.^8^ **b** We selected one gene (*Mtdh*) to illustrate the distribution of maternal (*X*-axis) and paternal (*Y*-axis) counts across 286 cells. The weighted allocation counts (green) are connected to their corresponding unique counts by a line in the scatter plot

A read that maps to one allele of one gene is a unique read. A read that maps uniquely to one gene but to both allelic copies is an allelic multi-read. A read that maps to multiple genes but only to one allele at each is a genomic multi-read. A read that maps to multiple genes and to both alleles of any of those genes is a complex multi-read. Contrary to our intuition, complex multi-reads convey information about allele-specific expression (Supplementary Fig. 1). We obtained unique reads and weighted allocation counts for each of 286 cells. The sequence reads include 2.5% genomic multi-reads, 59.3% allelic multi-reads, and 23.3% complex multi-reads. Thus, the unique-reads method retains only 14.9% of the available reads for analysis. This substantial loss of information could lead to high variability of allelic proportions. As a result, we find that the unique-reads method finds more monoallelic expression (Fig. 2a and Supplementary Fig. 1), calling on average $\sim$66 more genes monoallelic in each cell. We also observed $\sim$1,908 genes where the unique-reads method fails to detect bi-allelic expression in some cells whereas weighted allocation counts are consistently bi-allelic, for example, *Mtdh* (Fig. 2b and Table 1). The high frequency of monoallelic expression calls from unique reads can be misinterpreted as allelic bursting and gene expression can appear to be more dynamic.

**Table 1:** Cross-tabulation (2$\times$2 table) of maternal and paternal allelic expression for *Mtdh* gene with unique reads and weighted allocation counts. The unique counts resulted in 88 cells with monoallelic expression while only seven monoallelic calls were seen with weighted allocation

Unique reads	Maternal allele	
	Not expressed	Expressed
Paternal allele		
Not expressed	56	39
Expressed	49	142

Weighted allocation	Maternal allele	
	Not expressed	Expressed
Paternal allele		
Not expressed	0	5
Expressed	2	279

### Partial pooling improves the accuracy of allelic proportions

Over the course of the embryonic time series, the frequency of allelic expression varies dramatically, especially in the earliest stages where there is a very high rate of maternal monoallelic expression. In order to avoid mixing very disparate cell types in our evaluation of partial pooling, we restricted our analysis to the 122 mature blastocyst cells, the largest group in Deng et al.^8^ data. These cells have average coverage of $\sim$14.8M reads per cell. This allowed us to down-sampled the data by randomly selecting 1% of reads to obtain an average coverage of 148k reads per cell — a depth of coverage similar to current scRNA-Seq applications. We estimated allelic proportions on both full and down-sampled data using each of four methods implemented in ${\mathtt{scBASE}}$. We compared the estimated allelic proportions from the down-sampled data to estimates obtained from the full data using the corresponding unique reads or weighted allocation estimates with no pooling. The full data are based on 100-fold more reads per sample and provide an approximate truth standard. A similar approach to evaluation of single-cell data analysis was employed by Huang et al.^19^.

**Fig. 3:**
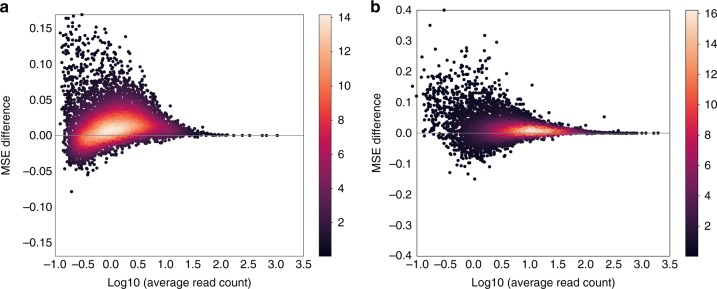
Partial pooling improves the accuracy of estimated allelic proportions. We randomly sampled 1% of reads from the full data of 122 mature blastocyst cells to obtain a sub-sample of 147,538 reads per cell, on average. We estimated gene- and cell-specific allelic proportions from the sub-sampled data, and computed mean squared error (MSE) between the estimated allelic proportions from the full data versus the sub-sampled data. We compared the MSE based on partial pooling versus the MSE from no pooling estimates, and display the difference (before $-$ after partial pooling) on the *y*-axis along the expression level in unique-read counts on the *x*-axis. We made this comparison for **a** unique reads and for **b** weighted allocation. Points representing individual genes are shown as a density heatmap

In order to assess the effects of partial pooling, we computed differences in the mean squared error (MSE) of estimated allelic proportions with and without partial pooling. Partial pooling applied to the unique-read counts improves estimation for the majority of genes (4,392 versus 1,367 out of 5,759 genes) with an average MSE difference of 0.018 (Fig. 3a). Partial pooling applied to the weighted allocation counts improves estimation for most genes (5,078 versus 1,673 out of 6,751 genes) with an average MSE difference of 0.016 (Fig. 3b). In both cases, the greatest gains are seen in the low expression range ($<10$ unique reads per gene). For the most highly expressed genes, there is little or no reduction in MSE, which is consistent with our expectation that pooling of information across cells is most impactful when coverage is low.

### The timing of allelic bursting is coordinated

**Fig. 4:**
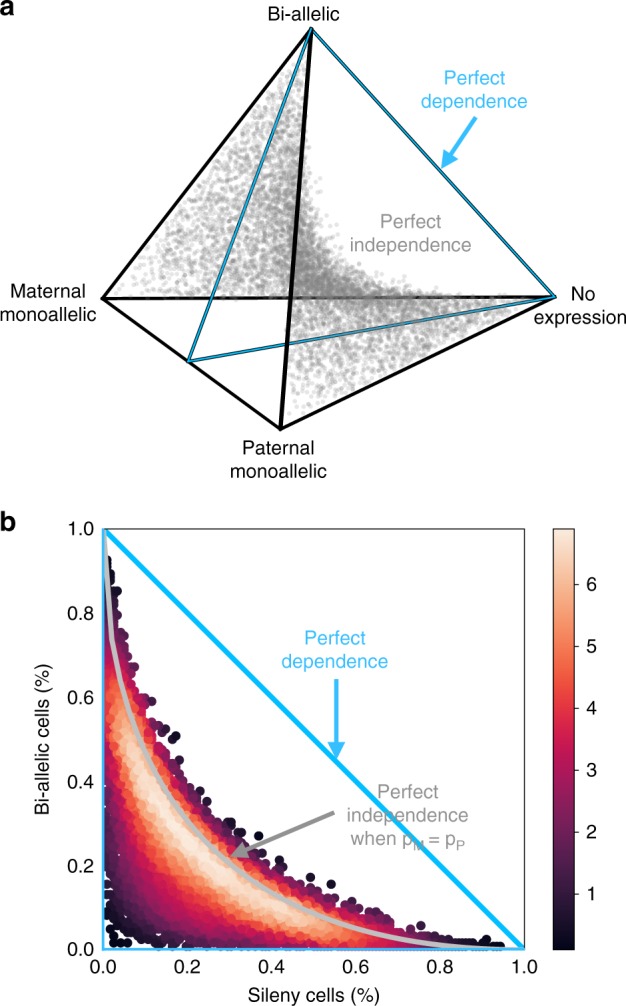
Independence of allelic bursting. **a** The geometry of the 2× 2 table proportions^23^ can be represented as a simplex, a 3D tetrahedral region of 4D space in which proportions are all non-negative and sum to one. The vertices of the simplex correspond to genes where all cells are in the same allelic expression state as indicated by labels. The distance from a vertex is inversely related (1-x) to the proportion of cells in that state. The point cloud inside the simplex represents random proportions according to the perfect independence model, i.e., the logOR equals zero. The blue triangle indicates proportions with equal maternal and paternal expression probability ${p}_{M}={p}_{P}$. **b** We simulated data under the perfect independence model without assuming ${p}_{M}={p}_{P}$ and plotted the proportions of bi-allelic and silent cells as in Deng et al.^8^

The timing of allelic bursting events is a defining feature of stochasticity in gene expression^20^. One fundamental question is whether the occurrence of allelic bursts is coordinated or if bursts occur independently for each allele. Statistical independence of maternal and paternal bursting can be evaluated using a $2\times 2$ table of counts of the numbers of cells for which a given gene is expressed only from the maternal allele, only from the paternal allele, from both, or not expressed (as in Table 1). If allelic bursts occur independently, the log-odds ratio (logOR) computed from this $2\times 2$ table should be close to zero. In order to relate this standard approach^21^ for testing the independence hypothesis to alternative methods^8,22^ that have been proposed for scRNA-Seq data, it is helpful to consider a geometric representation of the proportions of cells in each allelic expression state (Fig. 4a). Proportions are numbers greater than or equal to zero that sum to one. They can be represented as a point in a 3-dimensional tetrahedron in 4-dimensional space — the 4D simplex^23^. When maternal and paternal bursting events occur independently, the proportions should fall near the 3-dimensional surface within the simplex where the logOR is equal to zero (the point cloud region in Fig. 4a). The method of testing independence used in Deng et al.^8^ and Larsson et al.^22^ imposes an additional constraint on the $2\times 2$ table proportions by assuming that the frequencies of maternal and paternal bursting events are equal (${p}_{M}={p}_{P}$). This constraint corresponds to a 2-dimensional cross-section of the simplex, indicated by the blue triangle in Fig. 4a. Projection of points in the 4D simplex onto this triangle produces the graphical representation used by Deng et al. (e.g., Fig. 4b). This illustrates how the Deng et al. method is a special case of the logOR test.

**Fig. 5:**
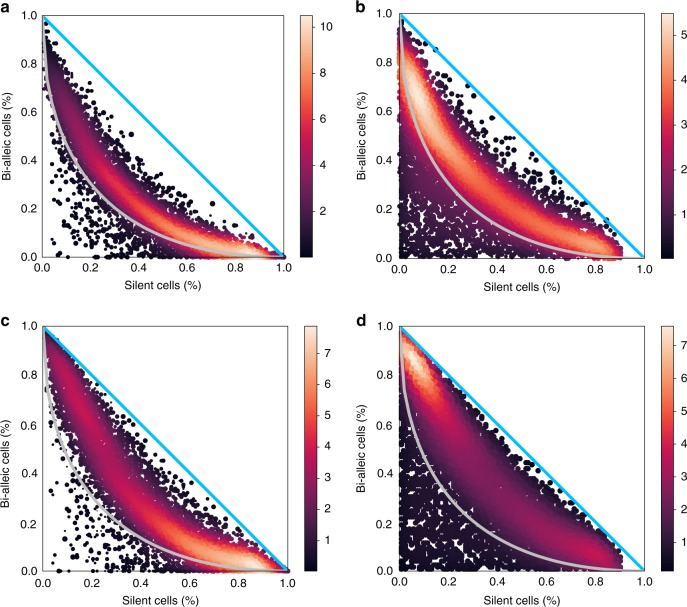
Independence of allelic bursting (continued). Four panels illustrate the proportions of bi-allelic and silent cells as estimated from **a** unique reads, **b** weighted allocation, **c** unique reads with partial pooling, and **d** weighted allocation with partial pooling. Points representing individual genes are shown as a density heatmap

We evaluated independence of allelic expression on the 122 mature blastocyst cells, as was done in Jiang et al.^24^. We first simulated data under the assumption of independent allelic bursting (Methods) and plotted the results to illustrate how points will be distributed in this diagram when the pure independence model is true (Fig. 4b). Next we estimated the $2\times 2$ table of allelic expression states using counts obtained from each of the four methods implemented in ${\mathtt{scBASE}}$. The appearance of the data in Fig. 5 is qualitatively distinct from the simulated data (Fig. 4b). Moreover, the null hypothesis of independence is rejected for the majority of genes regardless of the method used to estimate the allelic states (Supplementary Fig. 2a, b, c, d).

We evaluated independence with ${\mathtt{SCALE}}$ software, as in Jiang et al.^24^ using both unique reads and weighted allocation counts as input. We found significant non-independence for 3,381 genes using unique reads and for 4,815 genes using weighted allocation. We then applied partial pooling and found 6,068 significant genes using unique reads with partial pooling and 6,761 significant genes using weighted allocation with partial pooling. These results are reported using a false discovery rate of 5%. To assess the magnitude of the departure from independence we note that 2,845 and 3,763 (out of 8290) genes had $|$logOR$| \, > \, 2$ using unique reads and weighted allocation, respectively. After partial pooling 5,622 and 6,209 genes have $|$logOR$| \, > \, 2$ for unique reads and weighted allocation, respectively. The majority of genes had positive logOR, indicating a tendency for bursting to occur more in synchrony than chance would predict (Supplementary Fig. 2e).

We repeated these analyses using three additional datasets^6,22,25^ and arrive at similar conclusions in each case (Supplementary Figs. 3, 4, 5, 6, 7, and 8). The evidence for statistical dependence of bursting is strong and application of weighted allocation and partial pooling strengthens this conclusion.

### Characterizing allelic imbalance across a cell population

**Fig. 6:**
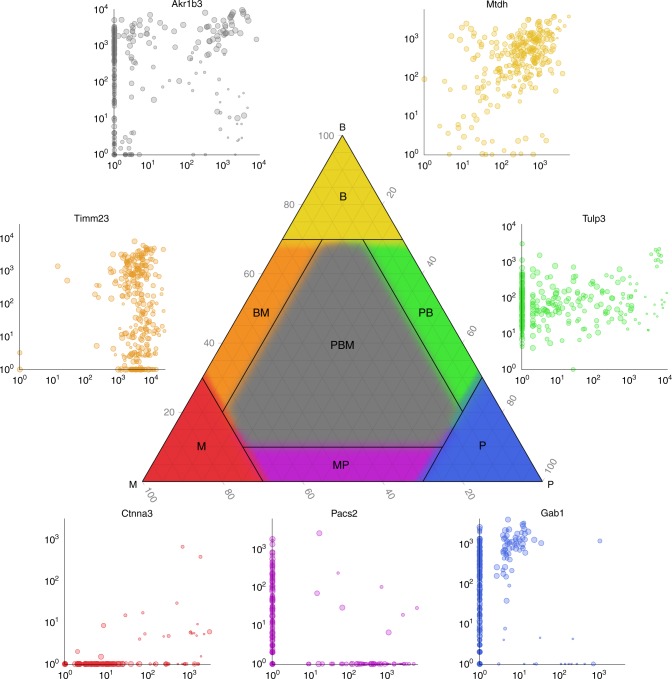
Classification of allele-specific expression patterns across cells. For each gene in each cell, the classification step of ${\mathtt{scBASE}}$ estimates allelic state probabilities ${\pi }_{gk}^{s}$, where $s$ indicates paternal monoallelic (P), bi-allelic (B), or maternal monoallelic (M) expression. The average proportions of cells in each allelic state (${\pi }_{g\cdot }^{s}$) can be represented as a point in a triangular diagram, which is a 3D simplex generated by projecting out the no-expression dimension from the 4D simplex in Fig. 4a. A gene that is predominantly paternal, bi-allelic, or maternal across the cell population will be plotted near the corresponding vertex. Points representing genes with mixed classification states across the cell population will appear along the edges or in the center of the triangle. We delineate seven patterns of allelic expression for a gene as indicated by the different colored regions in the diagram: ${\bf{P}}$ (blue), ${\bf{B}}$ (yellow), ${\bf{M}}$ (red), ${\bf{PB}}$ (green), ${\bf{BM}}$ (orange), ${\bf{MP}}$ (purple), and ${\bf{PBM}}$ (gray). Examples of genes from each pattern are shown as scatter plots of maternal and paternal read counts (log10 scale). Each point in the scatter plot corresponds to one cell (*n* = 286 embryo cells). For example, the gene *Pacs2* is expressed from either the maternal or the paternal allele but rarely both and is classified as an ${\bf{MP}}$ gene. The bi-allelic region (${\bf{B}}$) includes genes that may show allelic imbalance (${p}_{gk}\, \ne \, \frac{1}{2}$) across many cells but consistently express both alleles (e.g., *Mtdh*). The ${\bf{PB}}$ and ${\bf{BM}}$ regions will include genes that show a mixture of bi-allelic expression and monoallelic expression. Many of the genes in these regions have strong allelic imbalance and cells with monoallelic expression could be due to statistical sampling zeros in the lower expressed allele (e.g., *Tmim23* and *Tulp3*). The expression pattern in blastocyst cells for the majority of genes (57%) fall in the ${\bf{PBM}}$ region and display a pattern that is a mix of mono- and bi-allelic expression states across cells (e.g., *Akr1b3*)

The ${\mathtt{scBASE}}$ classification step provides a way to characterize the distribution of allelic expression states for any gene across a population of cells. We first compute the posterior probability of allelic expression states P, B, and M, for each gene in each cell. This classification assumes that all genes are expressed at some level, which may be very low for some genes. Thus, there is no state representing the absence of expression. This allows us to classify the allelic expression of cells that may have zero read counts due to statistical sampling. For each gene, we then estimate the proportion of cells in each allelic expression state and represent these proportions as points in a triangular simplex diagram. (Note that this representation is coming from projecting out the no-expression dimension in the 4D simplex, Fig. 4a.) To interpret the distribution of allelic expression across cells, we designate seven patterns of allelic expression (Fig. 6). Genes that are predominantly expressed as P, B, or M will appear near the corresponding vertex of the triangle (${\bf{P}}$, ${\bf{B}}$ or ${\bf{M}}$ region). Genes with mixed allelic states will appear along the edges (${\bf{PB}}$, ${\bf{BM}}$, or ${\bf{MP}}$ region) or near the center of the triangle (all three states, ${\bf{PBM}}$ region). For example, the gene *Pacs2*, which is expressed from either the maternal or the paternal allele but rarely both, is classified as an ${\bf{MP}}$ gene. The bi-allelic region (${\bf{B}}$) includes genes that are consistently expressed from both alleles e.g., *Mtdh*. The ${\bf{PB}}$ and ${\bf{BM}}$ regions include genes that show a mixture of bi-allelic and monoallelic expression with a strong allelic imbalance, e.g., *Timm23* and *Tulp3*. The majority of genes (56.9%) in the blastocyst stages of development are in the ${\bf{PBM}}$ region (Supplementary Fig. 9). These genes display a mix of mono- and bi-allelic expression states (e.g., *Akr1b3*) that is consistent with dynamic bursting of allele-specific gene expression.

**Fig. 7:**
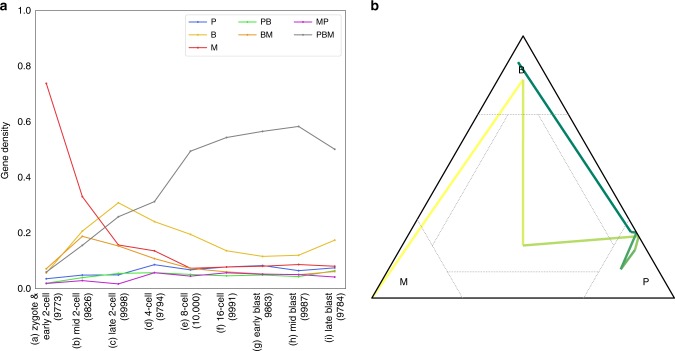
Dynamics of allele-specific expression patterns along the embryo development. **a** Cells were divided into nine developmental stages as indicated on the *X*-axis. The cell types and numbers of expressed genes at each stage are indicated in parentheses on the *X*-axis. For each stage, we counted the proportion of expressed genes that fall into each of the seven allelic expression patterns (*Y*-axis), indicated by lines using the same color coding used in Fig. 6. In the zygote and early 2-cell stage, most genes show purely maternal expression (${\bf{M}}$). The proportion of maternally expressed genes decreases through subsequent stages of development. The numbers of genes showing purely paternal expression (${\bf{P}}$) is low across all developmental stages. The ${\bf{M}}$ and ${\bf{P}}$ classes become equally represented in the later stages of development. The 2- and 4-cell stages show high levels of bi-allelic expression (${\bf{B}}$) and the mixed class (${\bf{PBM}}$) proportion becomes highest by the 8-cell stage. **b** The expected proportions of cells in each allelic state (${\pi }_{g\cdot }^{s}$) for one gene *Akr1b3* at each stage of the developmental time course is shown as a trajectory in the 3D simplex. Yellow to blue color line segments indicates the transitions between developmental stages. This gene starts in the maternal monoallelic state (${\bf{M}}$), it transitions through ${\bf{PBM}}$ to a paternal expression state (${\bf{P}}$), and then transitions to bi-allelic expression (${\bf{B}}$) in the blastocyst stages

We applied ${\mathtt{scBASE}}$ with weighted allocation and partial pooling to track changes in the ASE patterns of cells sampled over a developmental time course (Fig. 7a, Supplementary Figs. 9 and 10). Our aim is to classify allelic state distributions within subpopulations of cells defined by developmental stages. To achieve this, we first ran ${\mathtt{scBASE}}$ MCMC algorithm on all 286 cells to estimate the prior parameters, ${\alpha }_{g}^{s}$ and ${\beta }_{g}^{s}$ (Fig. 1 and Supplementary Methods). These parameters describe the distribution of allelic proportions in each allelic state. We then ran ${\mathtt{scBASE}}$ EM algorithm (with the prior parameters fixed) on each subpopulation of cells to estimate developmental stage-specific parameters (see Methods). In the zygote and early 2-cell stages, most genes show monoallelic maternal expression. At this stage, the hybrid embryo genome is not being transcribed and the mRNA present is derived from the mother (inbred CAST genome). At the mid 2-cell stage the hybrid embryo is being transcribed and we start to see expression of the paternal allele for some genes. Many genes exhibit the ${\bf{M}}$ and ${\bf{BM}}$ patterns through the 8- or 16-cell stages perhaps due to the persistence of long-lived mRNA species that were present at the 2-cell stage. The bi-allelic class ${\bf{B}}$ dominates the late 2-cell and 4-cell stages indicating high levels of expression at rates that exceed the half-life of most mRNA species. In the later stages of development, 8-cell through late blastocyst, most genes transition into the ${\bf{PBM}}$ pattern.

There are $\sim \! 400$ genes that make dramatic transitions across allelic expression states. For example, *Akr1b3* (Fig. 7b) starts in the zygote and early 2-cell stage with only maternal alleles present. It transitions to bi-allelic expression by the mid 2-cell stage indicating the onset of transcription of the paternal allele. It then transitions through the paternal monoallelic state. Our interpretation is that the early maternally derived transcripts were present prior to fertilization and these transcripts are still present when the paternal allele in the hybrid embryo gene starts to express. The early maternal transcripts are largely degraded by the 4- to 8-cell stages where we see only expression from the paternal allele. In the early blastocyst stages, we start to see embryonic expression of maternal alleles resulting in a bi-allelic expression pattern by the late blastocyst stage.

## Discussion

Allelic expression in single cells has provided new insights into the dynamic regulation of gene expression^6^. However, estimates of allelic proportions can display high statistical variation due to low depth of sequencing coverage per cell. The common practice of discarding multi-mapping reads exacerbates this problem. The ${\mathtt{scBASE}}$ algorithm reduces statistical variability by retaining and disambiguating multi-read data. It further improves estimation of allelic proportions by partial pooling of information across cells in the same ASE states. As a result we can obtain a more precise and accurate picture of gene expression dynamics in which biological stochasticity is revealed by reducing statistical variation.

Weighted allocation has been demonstrated to improve gene expression estimation in whole-tissue RNA-Seq^15–17^. When estimating total gene expression with weighted allocation, only genomic multi-reads need to be resolved and these typically represent a small proportion of all reads. When estimating allele-specific expression, however, depending on the levels of nucleotide heterozygosity, the majority of reads may lack distinguishing polymorphisms and will be allelic multi-reads. Complex multi-reads with ambiguity in both genomic and allelic alignment can carry useful information about allele-specific expression, as illustrated in Supplementary Fig. 1.

${\mathtt{scBASE}}$ uses partial pooling in the context of a mixture model with three allelic expression states (paternal monoallelic, bi-allelic, and maternal monoallelic) to preserve cell-to-cell heterogeneity by pooling information across cells that are in the same state. Combining information across cells, therefore, does not weaken the signals of strong allelic imbalance. We applied ${\mathtt{scBASE}}$ to X chromosome genes in female cells of three different datasets^6,8,25^. In the Reinius et al. fibroblast data, partial pooling corrected the allelic proportions of *Xist* gene expression towards either maternal or paternal monoallelic expression for both unique reads and weighted allocation counts (Supplementary Fig. 11). Looking at expression of all X chromosome genes in these same cells, we observe that partial pooling strengthens the expected pattern of expression due to X chromosome inactivation (XCI) consistent with *Xist* allele expression (Supplementary Fig. 12). We observe that XCI is often incomplete and not uniform across cells. In the Chen et al. and Deng et al. datasets, *Xist* is clearly in the bi-allelic expression state in many of mouse embryo cells, epistem cells, or motor neuron cells and this is preserved after partial pooling (Supplementary Figs. 13 and 14). We also observe that XCI is not fully established for these cells (Supplementary Figs. 15 and 16). In addition, for genes that are reported to be imprinted^26–28^ we examined their allelic expression. Irrespective of the estimation method applied, many of these genes do not appear to be fully imprinted in these three datasets (Supplementary Figs. 17, 18, 19, and 20). However, for those genes that do show evidence of imprinting, i.e., appear in ${\bf{M}}$- or ${\bf{P}}$-class, partial pooling improves the evidence for monoallelic expression for both unique reads and weighted allocation counts.

The ${\mathtt{scBASE}}$ analysis incorporates statistical uncertainty in both the classification of allelic expression state and the estimated allelic proportions of a gene. To evaluate the precision of the estimated parameters, we have computed the posterior standard deviation of allele proportions across a range of total read counts and with varying numbers of cells (286 cells versus 60 cells). The trends are as expected, deeper read coverage or more cells improves the precision of estimation (Supplementary Fig. 21). Our probabilistic classification accounts for uncertainty and can estimate the allelic expression state of a gene even when few or no reads are sampled from a given cell based on the behavior of other cells. The ${\mathtt{scBASE}}$ model is still reliable with degenerate inputs, for example, in the most extreme case of a single cell and a gene with zero total reads, the algorithm provides a sensible answer: class probabilities are $(\frac{1}{3},\frac{1}{3},\frac{1}{3})$ and a nearly uniform distribution for allelic proportion (mean at 0.5 with standard deviation of 0.2), indicating that the data do not contain any information. As the number of cells or the read depth increases, the class probabilities become more concentrated and the posterior distribution for the allelic proportion gets narrower. Partial pooling has the biggest impact when read coverage is low and the number of cells is large (Fig. 3 and Supplementary Fig. 21).

${\mathtt{scBASE}}$ software can be implemented as part of a scRNA-Seq analysis pipeline. For example, we ran ${\mathtt{SCALE}}$ software, which analyzes the dynamics of gene expression, using ${\mathtt{scBASE}}$ estimated counts as input. We found that both weighted allocation and partial pooling counts identified many more genes as non-independent (Results and Supplementary Figs. 2a, b, c, d). Our findings suggest that running ${\mathtt{SCALE}}$ with ${\mathtt{scBASE}}$ estimated counts as input will result in more accurate estimates of bursting kinetics and reduced levels of monoallelic gene expression when compared to standard pipelines that rely on unique-read counts.

The statistical properties of allelic bursting shed light on the nature of gene expression regulation. If expression bursts are statistically independent, this would imply that the regulation of allelic expression is local and acting autonomously at each allele. Under the perfect independence model, there would be no shared regulation of expression across alleles and the counts of cells in each allelic state will satisfy statistical criteria for independence. Under an alternative model, perfect dependence, bursting would be precisely coordinated across alleles and bursts would occur synchronously. All cells would be in either the bi-allelic or not expressed states. Our analysis of published scRNA-Seq data from four different experiments^6,8,22,25^ indicates that neither of these extremes is true (Fig. 5 and Supplementary Figs. 2, 3, 4, 5, 6, 7, and 8). We observed that the pattern of bursting is statistically dependent and positively correlated (${\mathrm{logOR}} \, > \, 0$) for the majority of genes. It is neither statistically independent nor perfectly synchronous. This suggests that regulation of allelic expression has both shared and locally autonomous components. While our statistical analysis cannot identify the mechanisms of regulation, it seems plausible that diffusible transcription factors could be responsible for the coordinated component of regulation. Local control is likely to be cis-acting and may involve stochastic variation in the activation of the transcriptional machinery. Additional experimental work would be required to test these hypotheses and to identify the cis-acting molecular events that trigger bursting of gene expression. However, the available data are sufficient to reject both hypotheses of perfect independence and of perfect dependence of allelic bursting.

Weighted allocation of multi-reads captures information in scRNA-Seq that is lost when multi-reads are discarded. It results in fewer spurious monoallelic expression calls and improves the accuracy of estimated allele proportions. Partial pooling is a technique for leveraging the information across many cells to improve the precision of estimation at the individual cell level. Pooling must be accomplished without compromising the cell-to-cell heterogeneity that single-cell analysis aims to reveal. In ${\mathtt{scBASE}}$, this is achieved by pooling within classes of a mixture model of allelic expression states. Based on the evaluations presented here, we recommend weighted allocation with partial pooling as the best approach to estimate expected counts for scRNA-Seq data. However, the ${\mathtt{scBASE}}$ software implements alternative methods, which could be useful for further evaluation in diverse applications. The retention of multi-mapping sequence reads and partial pooling of information are approaches that can be applied to a wide range of sequencing applications but they are especially critical in single-cell analysis where the number of cells is large and the number of reads available to quantify each gene in each cell may be very small.

## Methods

### Data

Deng et al.^8^ sampled 286 pre-implantation embryo cells from an F1 hybrid of CAST$\times$B6 along the stages of prenatal development. Embryos were manually dissociated into single cells using Invitrogen TrypLE and single-end RNA-Seq sequencing was performed using Illumina HiSeq 2000 (Platform GPL12112). There were fastq-format read files for four single-cell samples from zygote stage, eight from early 2-cell, 12 from mid 2-cell, 10 from late 2-cell, 14 from 4-cell, 47 from 8-cell, 30 from 16-cell, 43 from early blastocyst, 60 from mid-blastocyst, and 58 from late blastocyst stage. The Reinius et al. data^6^ consist of primary mouse fibroblast cells from the F1 reciprocal crosses of CAST$\times$B6 (125 cells, sex-typed) and B6$\times$CAST (113 cells, sex-typed). The Chen et al. data^25^ are from mouse embryonic stem cells (mESCs) from an F1 hybrid of B6$\times$CAST: 111 mESCs cultured in 2i and LIF, 120 mESCs cultured in serum and LIF, 183 mouse Epistem cells (mEpiSCs), and 74 post-mitotic neuron cells. The samples are sex-typed. Larsson et al.^22^ generated 224 individual primary mouse fibroblast cells from the F1 hybrid of CAST$\times$B6. As the data are from non-standard SMART-Seq2 platform, we downloaded the allele-specific UMI counts directly from their ${\mathtt{txburst}}$ github repository (https://github.com/sandberg-lab/txburst), and did not apply weighted allocation to these data. See Data Availability below.

### scRNA-Seq read alignment

For the F1 hybrid mouse we aligned reads to a phase-known diploid transcriptome — this is a best-case scenario for phasing. When dealing with more complex genomes, phasing should be performed beforehand if haplotype-specific transcriptomes are not available and ${\mathtt{scphaser}}$^29^ is one possible approach. We reconstructed the CAST genome by incorporating known SNPs and short indels (Sanger REL-1505) into the reference mouse genome sequence (Genome Reference Consortium Mouse Reference 38) using ${\mathtt{g2gtools}}$ (http://churchill-lab.github.io/g2gtools/). We lifted the reference gene annotation (Ensembl Release 78) over to the CAST genome coordinates, and derived a CAST-specific transcriptome. The B6 transcriptome is based on the mouse reference genome. We constructed a bowtie (v1.0.0) index to represent the diploid transcriptome with two alleles of each transcript. We aligned reads using bowtie with parameters ‘–all’, ‘–best’, and ‘–strata’, allowing for three mismatches (‘-v 3’). These settings enable us to find all of the best alignments for each read. For example, if there is a zero-mismatch alignment for a read, all alignments with zero-mismatch will be accepted.

### Overview of the scBASE model

The ${\mathtt{scBASE}}$ algorithm is composed of three steps: read counting, classification, and partial pooling (Fig. 1). The read counting step is applied first to resolve read mapping ambiguity due to multi-reads and to estimate expected read counts. The read counting step is not a requirement since the following steps are applicable to any allele-specific count estimates. The classification and partial pooling steps are executed iteratively to classify the allelic expression state and to estimate the allelic proportions for each gene in each cell using a hierarchical mixture model. We have implemented ${\mathtt{scBASE}}$ as a Monte Carlo Markov chain (MCMC) algorithm^30^, which randomly samples parameter values from their conditional posterior distributions. We have also implemented the classification and partial pooling steps as an Expectation-Maximization (EM) algorithm^31^ that converges to the maximum a posteriori parameter estimates (Supplementary Methods). MCMC is flexible, and the sampling distributions and priors are easy to change in the MCMC code. MCMC provides the full posterior distribution of allelic proportions and thus provides useful information about the uncertainty of estimated parameters. We also found that MCMC is more stable when fitting allelic proportion of monoallelic classes. The EM algorithm is much faster, but it provides only point estimation. We provide a brief description of the algorithm here and provide additional details in Supplementary Methods.

*Read counting*: In order to count all of the available sequence reads for each gene and allele, we have to resolve read mapping ambiguity that occur when aligning reads to a diploid genome. Genomic multi-reads align with equal quality to more than one gene. Allelic multi-reads align with equal quality to both alleles of a gene. In ${\mathtt{scBASE}}$, multi-reads are resolved by computing a weighted allocation based on the estimated probability of each alignment. We use an EM algorithm implemented in ${\mathtt{EMASE}}$ software for this step^17^. Alternatively, read counting could be performed using similar methods implemented in ${\mathtt{RSEM}}$^15^ or ${\mathtt{kallisto}}$^16^ software. The estimated maternal read count (${x}_{gk}$) for each gene ($g$) in each cell ($k$) is the weighted sum of all reads that align to the maternal allele, where the weights are proportional to the probability of the read alignment. Similarly, the estimated paternal read count (${y}_{gk}$) is the weighted sum of all reads that align to the paternal allele. The total read count is the sum of the allele-specific counts (${n}_{gk}={x}_{gk}+{y}_{gk}$). A parameter of interest is the allelic proportion ${p}_{gk}$. The read counting step provides an initial estimate ${\hat{p}}_{gk}={x}_{gk}/{n}_{gk}$, which we refer to as the weighted allocation estimated counts (b).

*Classification*: In the classification step, we estimate the allelic expression state (${z}_{gk}$) for each gene in each cell. The allelic expression state is a latent variable with three possible values ${z}_{gk}\in \{P,B,M\}$ representing paternal monoallelic, bi-allelic, and maternal monoallelic expression, respectively. Uncertainty about the allelic expression state derives from sampling variation that can produce zero counts for one or both alleles even when the allele-specific transcripts may be present in the cell. We account for this uncertainty by computing a probabilistic classification based on a mixture model in which the maternal read counts ${x}_{gk}$ are drawn from one of three beta-binomial distributions (given ${n}_{gk}$) according to the allelic expression state ${z}_{gk}$. For a gene in the bi-allelic expression state the maternal allelic proportion is denoted ${p}_{gk}^{B}$ and, as suggested by the notation, it may vary from cell to cell following a beta distribution. For a gene in the paternal monoallelic expression state, the allelic proportion ${p}_{g}^{P}$ follows a beta distribution with a high concentration of mass near zero. Similarly, for a gene in the maternal monoallelic expression state, we model ${p}_{g}^{M}$ using a beta distribution with the concentration of mass near one. The beta distribution parameters for the maternal and paternal states are gene-specific but are constant across cells.

*Partial pooling*: The classification step assumes that the mixture model parameters are known. This model describes gene-specific allelic proportions for each cell and thus it has a very large number of parameters. In the scRNA-Seq setting where thousands of genes are measured but low read counts and sampling zeros are prevalent, we may have limited data to support their reliable estimation. Bayesian analysis using a hierarchical model is well suited for estimation in settings with large numbers of parameters. In this context, the hierarchical model improves the precision of estimation by borrowing information across cells for each gene, giving more weight to cells that are in the same allelic expression state. This estimation technique is referred to as partial pooling. Specifically, we sample the mixture weights $({\pi }_{g\cdot }^{P},{\pi }_{g\cdot }^{B},{\pi }_{g\cdot }^{M})$ and the class-specific allele proportions $({p}_{g}^{P},{p}_{gk}^{B},{p}_{g}^{M})$; generate classification probabilities $({\pi }_{gk}^{P},{\pi }_{gk}^{B},{\pi }_{gk}^{M})$; and then estimate the allelic proportions as a weighted average

1 $${p}_{gk}={\pi }_{gk}^{P}\ {p}_{g}^{P}+{\pi }_{gk}^{B}\ {p}_{gk}^{B}+{\pi }_{gk}^{M}\ {p}_{g}^{M}$$

The average value across many iterations is ${\tilde{p}}_{gk}$, the partial pooling estimator.

### Estimating allelic proportions in subsets of cells or genes

The ${\mathtt{scBASE}}$ algorithm is designed to model heterogeneous ASE states in any population of cells. In some experiments, we may be interested in the distribution of allelic expressions states within pre-defined groups of cells. For example, in the data from Deng et al., the early stages of development are highly skewed toward maternal expression. If the groups of cells are large, we could apply ${\mathtt{scBASE}}$ separately for each group. However, if the the number of cells is small, we recommend a two-stage procedure. First, run MCMC with all of the available cells to estimate the prior parameters, ${\alpha }_{g}^{s}$ and ${\beta }_{g}^{s}$. These prior parameters are common across all cells and we can estimate them most accurately in this way. Then, holding ${\alpha }_{g}^{s}$ and ${\beta }_{g}^{s}$ fixed, re-estimate the group-specific parameters, ${\pi }_{g\cdot }^{s}$, ${\pi }_{gk}^{s}$, and ${p}_{gk}$, within each cell type using the EM algorithm version of ${\mathtt{scBASE}}$. We applied this approach to Deng et al. data to generate Fig. 7a.

When groups of genes are expected to have different distributions of allelic states, e.g., X chromosome genes, it makes sense to run ${\mathtt{scBASE}}$ separately for these genes. Our analyses of female X chromosome genes used this strategy (Supplementary Figs. 12, 15, 16).

### Assigning allelic expression states from estimated counts

Unique-read counts are obtained directly from counting reads after discarding all genomic and allelic multi-reads. Weighted allocation counts are derived from the EM algorithm as described above. To estimate counts after partial pooling, we multiply ${\tilde{p}}_{gk}$ by the total gene expression counts. We note that estimated counts are not integers and may be non-zero but less than one. Classification of allelic expression states for each gene in each cell directly from observed or estimated counts requires setting a threshold for monoallelic expression. For each allele, we regarded it as expressed if its estimated abundance is greater than one reads (or one UMI as in Larsson et al.^22^).

### Gene classification using its ASE profile across many cells

We classify a gene according to the proportion of cells in P-, B-, and M-states, $({\pi }_{g\cdot }^{P},{\pi }_{g\cdot }^{B},{\pi }_{g\cdot }^{M})$, that are estimated by the partial pooling model. If a majority of cells (${\pi }_{g\cdot }^{s}\, > \, {0.7}$) are in a particular ASE state, $s\in \{P,B,M\}$, then we will assign the gene to the class ${\bf{P}}$ (monoallelic paternal; blue), ${\bf{B}}$ (bi-allelic; yellow), or ${\bf{M}}$ (monoallelic maternal; red) respectively. When a majority of cells are a mixture of two of those classes (${\pi }_{g\cdot }^{{s}_{1}}+{\pi }_{g\cdot }^{{s}_{2}}\, > \, {0.9}$ where ${s}_{1},{s}_{2}\in \{P,B,M\}$), we classify it into either of ${\bf{PB}}$ (mixture of monoallelic paternal and bi-allelic; green), ${\bf{BM}}$ (mixture of monoallelic maternal and bi-allelic; orange), or ${\bf{MP}}$ (a mixture of monoallelic maternal and paternal; purple). Otherwise, genes that present all three ASE states are classified as ${\bf{PBM}}$ (mixture of all; gray). We specified these seven classes in a ternary simplex diagram^32^ (Fig. 6). The class boundaries are arbitrary but the aim of this classification is to provide a simple descriptive summary of the gene expression states present in a population of cells.

### Sampling reads

We randomly sampled 1% of reads in each of 122 cells at the early, mid, and late blastocyst stages to obtain an average read count of $\sim$148k reads per cell. We chose the blastocyst cell types because, unlike cells in earlier developmental stages, they show the widest range of different states of allelic expression. The original analysis of ${\mathtt{SCALE}}$^24^ also used the same 122 cells. We applied the unique-reads method and weighted allocation algorithm to the full set of $\sim$14.8M reads and also applied each of four estimation methods (unique reads, weighted allocation counts, unique reads with partial pooling, and weighted allocation with partial pooling) to the down-sampled data. We compared estimates obtained from the down-sampled data to the full data estimates and computed the mean squared error of estimation across cells for each gene.

### Simulation of counts under perfect independence model

We randomly sampled the marginal probabilities of maternal and paternal allelic expression, ${p}_{M}$ and ${p}_{P}$ from uniform distribution between 0 and 1. Then we generated 2$\times$2 tables by sampling counts from multinomial distribution with probability $\{{p}_{M}{p}_{P},\ \ \ {p}_{M}(1-{p}_{P}),\ \ \ (1-{p}_{M}){p}_{P},\ \ \ (1-{p}_{M})(1-{p}_{P})\}$ for bi-allelic, maternal monoallelic, paternal monoallelic, and silent cells respectively.

### Reporting summary

Further information on research design is available in the Nature Research Reporting Summary linked to this article.

## Supplementary information

Reporting Summary

Supplementary Informatioin

Peer Review

## Data Availability

We downloaded Deng et al.^8^ data, Series GSE45719, from Gene Expression Omnibus (GEO) at http://www.ncbi.nlm.nih.gov/geo/query/acc.cgi?acc=GSE45719. Reinius et al.^6^ data are available from GEO at https://www.ncbi.nlm.nih.gov/geo/query/acc.cgi?acc=GSE75659. We downloaded Chen et al.^25^ data (files in SRA format) available at https://www.ncbi.nlm.nih.gov/geo/query/acc.cgi?acc=GSE74155. For the analysis of Larsson et al.^22^ data, we downloaded the allele-specific UMI counts from https://github.com/sandberg-lab/txburst/tree/master/data (as of 19 April 2019). All other relevant data are available upon request.

## Code availability

We have implemented our approach in extensible open-source software, scBASE, available at https://github.com/churchill-lab/scBASE with the MIT licence.
