# Supplementary material for: A Bayesian mixture model for the analysis of allelic expression in single cells
Source: Nat Commun. 2019 Nov 15;10:5188. doi: 10.1038/s41467-019-13099-0 (PMC6858378; doi:10.1038/s41467-019-13099-0)
Supplement: Supplementary file 3 — Peer Review [file 41467_2019_13099_MOESM3_ESM.pdf]

**Editorial Note:** Parts of this peer review file have been redacted as indicated to remove third-party material where no permission to publish could be obtained.

Reviewers' comments:

Reviewer #1 (Remarks to the Author):

Single-cell RNA-seq provides a unique opportunity to study the dynamics of allelic gene expression. Although there have been many scRNA-seq studies, the available statistical methods to analyze such data for allelic gene expression are still limited. So far, there is only one paper by Jiang et al. (2017 Genome Biology) that systematically studied allelic gene expression using scRNA-seq data. The paper by Choi et al. presented another statistical method to address this important, but under investigated problem.

A major contribution of the paper is the weighted allocation of multi-reads, which are mapped to multiple locations in the genome with similar sequence features. The authors used EMASE, an EM based algorithm to allocate multi-reads to the corresponding allele of each gene. After allele-specific reads are estimated, the authors then used a hierarchical mixture model to classify the allelic expression state for each gene in each cell. Using this hierarchical mixture model, with weighted multi-reads estimated allelic counts as input, the authors analyzed a scRNA-seq data generated by Deng et al. (2015 Science), which were also analyzed by Jiang et al. (2017). In contrast to Jiang et al., who found that most genes show independence between transcription of two alleles, the authors found that the transcription of two alleles tends to be coordinated, and such discrepancy is likely due to the use of those multi-reads, which were ignored by Jiang et al..

This paper is addressing an important problem, the methods are statistically rigorous, and the findings from the analysis of the Deng et al. dataset is also interesting. Below, I list a few comments, which I hope the authors could address to further improve the manuscript.

1. It is important to provide additional evidence to show that the multi-reads allocation gives reliable estimates of allele-specific counts. The authors illustrated the idea of multi-reads allocation in Figure 3. Given that the conclusion of the Deng et al. dataset heavily relies on the reliability of multi-reads allocation, it would be useful if the authors could provide additional evidence, either using an approach different from that shown in Figure 3, or analysis of a different dataset, to show that the proposed multi-reads allocation does give reliable allelic read counts compared to approaches that only use uniquely aligned reads.

2. The dynamic pattern shown in Figure 6 is quite interesting. One potential problem of this analysis is that some of the developmental stages have small number of cells; for example, zygote & early 2-cell, mid 2-cell, late 2-cell, and 4-cell stages, all have less than 20 cells. With such a small sample size, how can you ensure that the parameters from your hierarchical mixture model are reliably estimated? Related to this question, the authors should provide evidence to show that the hierarchical mixture model provides reliable results. This could be done through simulations. It would be useful to know how many cells and how many reads are needed to reliably estimate the parameters.

3. Deng et al. data didn’t use UMIs, thus the data could be biased by amplification bias. The current model didn’t consider amplification bias and dropout events, which cannot be ignored in non-UMI based scRNA-seq studies. How could these influence your conclusion? Is it possible to extend your model to account for amplification bias and dropout events? It would be nice if you could do that, as this will make your method more realistic.

4. Other than the use of multi-reads, what are the major differences between scBASE and Jiang et al.’s method? Since both papers analyzed the same dataset, it is worth comparing the results obtained

from these two methods in more detail.

Reviewer #2 (Remarks to the Author):

Choi et.al. have come up a new algorithm for the analysis of allelic expression in single cells. The difference from previous work is to use multi-reads and execute iteratively classification and estimation. The authors claimed that scBASE resulted in much lower false positive monoallelic calling. Such effort is valuable to the community but I find the work is relatively poorly done and is more suitable for specialized journals. My major points are followings:

1. It is confusing what cells the authors have used for analysis. At the beginning of the results, they mentioned that they restricted to 32 out of 286 for at least 4 allelic unique reads. But when they downsampled data to test sequencing depth for the algorithm, they used 60 cells. For figure 2b, they used all 286 cells. It would be good to make it clear what cell identity (i.e. stage) they used and why for each analysis.
2. In figure2a, it is not clear to me if genes checked for zygotes and 2-cells are also among those 13,006 genes. I supposed this graph is showing genes but not cells. Therefore, it is confusing to see the figure legend mentioning the outliners are cells (also what are these cells' identity?). The author claimed that using unique allelic reads inflated monoallelic calling with average 377 more genes. But I wonder why there is no such inflation for zygotes and 2-cells, which genes are maternally monoallelic expressed. Does this have something to do with the limited number of cells used for this analysis?
3. I wonder how scBASE correction affect allelic calling at different gene expression level. Allelic gene expression happens more often in the low-median expressed level of genes. How this method performs as the function of the expression level? They showcased one gene Mtdh. It would be good to systematically evaluate all genes.
4. I totally didn't get how the authors could use logOR to evaluate allelic burst and came to the conclusion that the burst occurred synchronously since more and more evidence points to other direction, which is independent bursting from two alleles (a publication is recently accepted by Nature from Rickard Sandberg's lab). The authors really need to have a positive control setting to prove this point such as looking at the allelic exclusion of the immunoglobulin heavy chain locus.
5. Figure 2 e and f evaluated how sequencing depths affected allelic calling, which is an important confounding factor. In this case, it would informative also to compare scBASE versus unique-reads methods.
6. The idea of partial pooling can overcome the problem of scare read counts. But one allelic bursting is often random and dynamic. When pooling, it was shown that allelic calling canceled out each other. It is not clear to me how the pooling will help for random allelic expression. Pooling will be only helpful if the gene is imprinted, which means that the allelic expression is consistent across all the cells.
7. I am not a mathematician. But I wonder how to benchmark the validity of weighted allocation to make sure that it doesn't introduce false positive as well since it is also based on possibility score. What is the parameter setting that can affect allelic calling? It would be great to discuss this.
8. As author also mentioned (ref 15) several allelic methods are available, such as scphaser (scphaser: haplotype inference using single-cell RNA-seq data), which takes advantage of the allelic calling nearby the reads. It is extremely important to benchmark scBASE with the published methods. Also, the data scale used is still very small. Recently, many data points are available with hybrid single-cell sequencing (e.g. Chen et.al Genome Research, PMID 27486082).

Reviewer #3 (Remarks to the Author):

In this study, the authors present a method, scBASE, to estimate Allele Specific Expression of a gene from single cell transcriptome data. Their method is supposed to deal with low coverage issues and allele dropout. Based on the presented data I find it difficult to evaluate the impact of the proposed algorithm on sc ASE analysis.
My main concern is that a significant part of the manuscript is based on the concept of multi-mapping reads. In the results they claim that « In a typical scRNA-seq workflow for ASE [..] only the unique 14.9% of the original sequence reads « are available for the analysis. And this result comes from the fact that 59.3% of reads have to be thrown away because «allelic multi-reads« i.e. not univocally mappable given the presence of (heterozygous) variances in the genome. It is quite difficult to comment on this statement. If that was true, dozens of papers on single cell transcriptomes should be retracted. But also the whole literature on genomic variations, eQTL and clinical genetic applications based on RNAseq and Whole Exome or Genome sequencing should be thrashed.
In reality, any aligner, including Bowtie, has no problem to assign a read to the proper allele (normally 90% of reads in RNAseq or scRNAseq experiments have perfect mapping quality and usable for the analysis) which makes all the first paragraph of the results meaningless. Are the authors capable to prove this statement wrong using BWA, Bowtie, Star and other aligners? Could they please show the distribution of mapping quality per read in the experiments they cite in the manuscript and prove that 85% of the reads are unusable for ASE analysis? What happen if all reads are used irrespective of their mapping quality?
If they cannot prove that, they need to show that the statistical recovery of the remaining 10% of reads (even less since many of the unmapped reads are not representing a biological signal) gives a significant improvement for ASE estimation.
The authors seem to criticize the usage of threshold ("arbitrary") in many other study but the whole manuscript is based on arbitrary choices:
1) What is the rationale of using a beta-binomial and half-Cauchy distributions with arbitrary parameters (2,7) as reported in the supplementary methods? (consider another study PMC5339288 using beta-binomial for ASE)
2) From where the authors derived the thresholds 0.7 and 0.9 in the paragraph "Classification of a gene according to itsASE profile across many cells"
3) From where the authors derived the thresholds 0.02 and 0.98 in the paragraph "calling monoallelic expression from no-pulling estimators of allelic proportion"
4) They claim that beta distribution parameters are gene specific. However they did not explained how these parameters change with respect to genes features. Gene length? GC content? Etc..

Downsampling the data to evaluate the performance of the method is a good idea.
5) The reference estimation should be performed with both weighted allocation and unique-reads (all of them) on the full dataset.
6) Really the authors removed 85% of the reads in the unique-reads approach when comparing their method? please perform the comparison using 1) all the reads 2) different thresholds of mapping quality
7) Could they validate their method with known imprinted genes and X inactivated genes during embryonic differentiation?
8) It is not clear when MCMC or EM to estimate pgk is used. What are the advantages of one approach over the other?

There are quite sparse statements about the dynamics of transcriptional bursting activity.
9) Could the authors make a specific paragraph and support with factual arguments statements such as "SCALE with scBASE […] will results in more accurate estimates of bursting kinetics" or "We also found that these data do not support the independence of allelic transcriptional bursts". In which genes the alleles are not independently transcribed? Is logOR sufficient to make this statement? No, if these genes are independently but frequently transcribed. What is the correlation between estimated

ASE and transcriptional level? Do their ASE correlate with half-life RNA?

**Reviewer #1 (Remarks to the Author):**

**Single-cell RNA-seq provides a unique opportunity to study the dynamics of allelic gene expression. Although there have been many scRNA-seq studies, the available statistical methods to analyze such data for allelic gene expression are still limited. So far, there is only one paper by Jiang et al. (2017 Genome Biology) that systematically studied allelic gene expression using scRNA-seq data. The paper by Choi et al. presented another statistical method to address this important, but under investigated problem.**
**A major contribution of the paper is the weighted allocation of multi-reads, which are mapped to multiple locations in the genome with similar sequence features. The authors used EMASE, an EM based algorithm to allocate multi-reads to the corresponding allele of each gene. After allele-specific reads are estimated, the authors then used a hierarchical mixture model to classify the allelic expression state for each gene in each cell. Using this hierarchical mixture model, with weighted multi-reads estimated allelic counts as input, the authors analyzed a scRNA-seq data generated by Deng et al. (2015 Science), which were also analyzed by Jiang et al. (2017). In contrast to Jiang et al., who found that most genes show independence between transcription of two alleles, the authors found that the transcription of two alleles tends to be coordinated, and such discrepancy is likely due to the use of those multi-reads, which were ignored by Jiang et al..**

**This paper is addressing an important problem, the methods are statistically rigorous, and the findings from the analysis of the Deng et al. dataset is also interesting. Below, I list a few comments, which I hope the authors could address to further improve the manuscript.**

**1. It is important to provide additional evidence to show that the multi-reads allocation gives reliable estimates of allele-specific counts. The authors illustrated the idea of multi-reads allocation in Figure 3. Given that the conclusion of the Deng et al. dataset heavily relies on the reliability of multi-reads allocation, it would be useful if the authors could provide additional evidence, either using an approach different from that shown in Figure 3, or analysis of a different dataset, to show that the proposed multi-reads allocation does give reliable allelic read counts compared to approaches that only use uniquely aligned reads.**

**Comment 1-1:** The problem of read alignment bias in allele-specific expression was first raised by Degner et al. (2009 Bioinformatics). Methods that employ an EM algorithm to compute weighted allocation of multi-reads have been carefully evaluated and are consistently the best options (Li & Dewey, 2011 BMC Bioinformatics; Munger et al., 2014 Genetics; Bray et al., 2016 Nature Biotech.; and Raghupathy et al, 2018 Bioinformatics). The examples presented in Figure 2 are intended to highlight the significant impact of weighted allocation in the context of single cell data. In light of extensive evaluation of the EM approach in the papers cited above, we chose to focus the remainder of this manuscript on other aspects of scBASE. However, see

(Comment 2-4) below in regard to independence of allelic bursting and (Comment 3-7) in regard to X chromosome and imprinted genes.

Although we strongly recommend using one of these weighted allocation methods, scBASE is agnostic to how allele-specific expression counts are derived. To clarify this point, we performed additional simulation tests and compared the accuracy of scBASE using either uniquely mapping read counts or weight allocated counts (new Figure 3, former Figure 2 (e)(f) but with revised results). The revised simulations demonstrate that combining information across cells improves the accuracy of estimated allele proportions for both weighted allocation and unique read counts especially in low expressed genes.

**2. The dynamic pattern shown in Figure 6 is quite interesting. One potential problem of this analysis is that some of the developmental stages have small number of cells; for example, zygote & early 2-cell, mid 2-cell, late 2-cell, and 4-cell stages, all have less than 20 cells. With such a small sample size, how can you ensure that the parameters from your hierarchical mixture model are reliably estimated? Related to this question, the authors should provide evidence to show that the hierarchical mixture model provides reliable results. This could be done through simulations. It would be useful to know how many cells and how many reads are needed to reliably estimate the parameters.**

**Comment 1-2:** The scBASE analysis incorporates statistical uncertainty (which is determined by read depth and number of cells) in both the classification of cells and the estimated allelic proportions. We failed to emphasize this point in the original submission. To address the question about reliability of the model parameters, we have now computed the posterior standard deviation of allele proportions across a range of total read counts and with varying numbers of cells (286 cells versus 60 cells). These data are summarized in new Supplemental Figure S4. The trends are as expected, deeper coverage or more cells improve the precision of estimation. It is difficult to make specific sample size recommendations without more context but we found that scBASE model is still reliable with degenerate inputs, for example, when data do not contain some ASE classes. Even in the most extreme case of a single cell and a gene with zero total reads, the algorithm provides a sensible answer: class probabilities are (⅓, ⅓, ⅓) and a nearly uniform distribution for allelic proportion (mean at 0.5 with standard deviation of 0.2), indicating that the data does not contain any information. As the number of cells or the read depth increases, the class probabilities become more concentrated and the posterior distribution for the allelic proportion gets narrower.

**3. Deng et al. data didn’t use UMIs, thus the data could be biased by amplification bias. The current model didn’t consider amplification bias and dropout events, which cannot be ignored in non-UMI based scRNA-seq studies. How could these influence your conclusion? Is it possible to extend your model to account for amplification bias and dropout events? It would be nice if you could do that, as this will make your method more realistic.**

**Comment 1-3:** Amplification bias is usually treated at the alignment level and should be addressed prior to scBASE analysis. Deng et al., did not specify whether their data were deduplicated, so we assumed that it was. With respect to extra zeros, we think the jury is still out. See two recent reports (https://f1000research.com/articles/7-1740/v1, https://www.biorxiv.org/content/early/2018/11/26/477794) regarding drawbacks of using zero-inflated models to represent dropout events. In fact, we implemented a zero-inflated version of scBASE and performance seemed to degrade. We felt that it was a bigger question than the scope of this manuscript, and have not carried out the extensive work that would be required to evaluate this question properly.

**4. Other than the use of multi-reads, what are the major differences between scBASE and Jiang et al.'s method? Since both papers analyzed the same dataset, it is worth comparing the results obtained from these two methods in more detail.**

**Comment 1-4:** The methods are different in their objectives. SCALE aims to quantify the dynamism of allelic bursting by parameterizing on and off state of each allele. scBASE aims to improve allelic proportion estimates in each cell by referring to the ASE state of the entire cell population. As we indicated the original discussion, scBASE output can be used as input data for SCALE. (Thanks for the reviewer's suggestion) We have now run SCALE analysis using counts based on unique-reads, weighted allocation, and scBASE. Our findings are summarized in new Supplemental Figure S6 and S7. We found that more genes (from 5,965 to 7,639 at FDR=5%) appeared to be non-independent when weighted allocation-based counts are used in SCALE. Even more genes (8,244) were identified as non-independent using counts based on scBASE. Although it is not mentioned in Jiang et al, substantial number (3,485 at FDR=5%) of genes were identified as non-independent in original analysis by Deng et al. (uniquely mapping reads). We concluded that independence hypothesis of allelic bursting is not supported by either scBASE or SCALE.

**Reviewer #2 (Remarks to the Author):**

**Choi et.al. have come up a new algorithm for the analysis of allelic expression in single cells. The difference from previous work is to use multi-reads and execute iteratively classification and estimation. The authors claimed that scBASE resulted in much lower false positive monoallelic calling. Such effort is valuable to the community but I find the work is relatively poorly done and is more suitable for specialized journals. My major points are followings:**

**1. It is confusing what cells the authors have used for analysis. At the beginning of the results, they mentioned that they restricted to 32 out of 286 for at least 4 allelic unique reads. But when they downsampled data to test sequencing depth for the algorithm, they used 60 cells. For figure 2b, they used all 286 cells. It would be good to make it clear what cell identity (i.e. stage) they used and why for each analysis.**

**Comment 2-1:** We used all 286 cells for our analysis. We restricted attention to the 13,006 genes that have at least 4 allelic unique reads in at least 32 of the cells. We used 60 mid-blastocyst cells for our simulation in which we down-sampled reads from the real data. We picked mid-blastocyst cell type because it was the largest uniform group of mature blastocyst cells and had high read depth (~13M per cell) that made them most suitable for our down-sampling simulation. In general, hierarchical models benefits when more cells are available, therefore using only 60 cells in simulations puts our model into a more challenging condition. We clarified the description in Line 288-292.

**2. In figure2a, it is not clear to me if genes checked for zygotes and 2-cells are also among those 13,006 genes. I supposed this graph is showing genes but not cells. Therefore, it is confusing to see the figure legend mentioning the outliers are cells (also what are these cells' identity?). The author claimed that using unique allelic reads inflated monoallelic calling with average 377 more genes. But I wonder why there is no such inflation for zygotes and 2-cells, which genes are maternally monoallelic expressed. Does this have something to do with the limited number of cells used for this analysis?**

**Comment 2-2:** Each data point in this figure represents a cell and we are showing all the 286 cells including zygote and 2-cells (highlighted). There is an inflation of maternal monoallelic genes in zygotes and 2-cells in the unique-reads method. We have now clarified this point in the figure caption (Line 371-376).

**3. I wonder how scBASE correction affect allelic calling at different gene expression level. Allelic gene expression happens more often in the low-median expressed level of genes. How this method performs as the function of the expression level? They showcased one gene Mtdh. It would be good to systematically evaluate all genes.**

**Comment 2-3:** As we described in the main text (Line 38-58, Figure 2, and Supplemental Figure S1), the quantification bias due to discarding multi-reads occurs in highly expressed genes too. To clarify this point, we looked into the results of SCALE based on unique-reads method and scBASE counts (See Figure R1(A) below). In this figure, there are a group of genes (along the y-axis) for which many more monoallelic cells are called using counts based on unique reads than with scBASE. When we plot the difference of monoallelic cell counts with same data along the burst sizes, we observe this group is not necessarily composed of low expressed genes (Figure R1(B)).

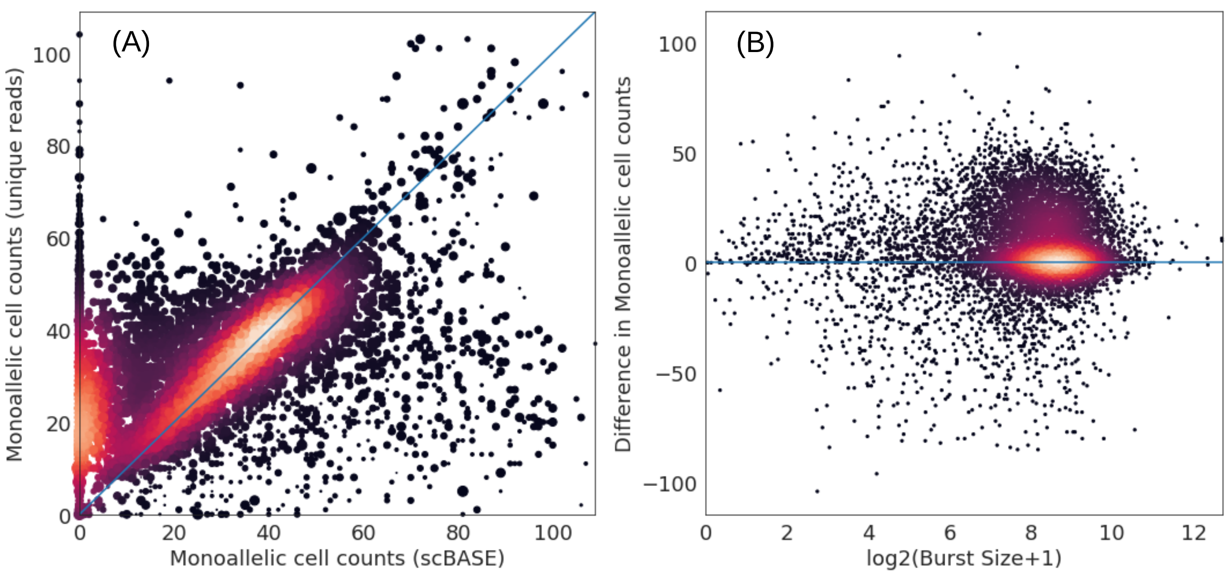


Figure R1: Monoallelic cell counts using counts based on unique reads and scBASE. Markers are proportionate to the estimated burst size of genes (A). Difference in monoallelic cell counts between estimation based on unique reads and scBASE. Data points in y>0 region are genes that unique-reads method reports more monoallelic cells (B).

In general, genes with low expression are most subject to miscalling ASE classes when we discard multi-reads. It is also in low expressed genes where statistical sampling is most likely to generate apparent monoallelic expression. The impact of scBASE is greatest on genes with low expression. This is where the biggest gains are achieved by combing information across cells in the same ASE states. Estimates for genes with high expression are not substantially changed by combining information across cells compared to the weighted allocation estimates based on individual cell data. This can be seen in our simulation tests (new Figure 3) as we have now commented in the revised manuscript (Line 70-81).

**4. I totally didn't get how the authors could use logOR to evaluate allelic burst and came to the conclusion that the burst occurred synchronously since more and more evidence points to other direction, which is independent bursting from two alleles (a publication is recently accepted by Nature from Rickard Sandberg's lab). The authors really need to**

**have a positive control setting to prove this point such as looking at the allelic exclusion of the immunoglobulin heavy chain locus.**

**Comment 2-4:** This question of statistical independence of bursting is important and we appreciate that the reviewer draws attention to it. We have carried out additional analyses to confirm that our conclusions are accurate. We stand by the odds ratio as the definitive standard for evaluating independence in a 2-way cross classification. In addition, we adopted the method used by Deng et al. (which imposes an unnecessary assumption that the marginal probabilities of maternal and paternal allele bursts are equal, $P_M$=$P_P$). We replicated the evaluation in Deng et al., and based on their own algorithm the evidence for statistical dependence is still strong. We then applied Deng's method to unique read counts and to weight allocated counts. The evidence for statistical dependence is strongest when we use weighted allocation to include the multi-reads and combine information across cells. These analyses are summarized in Line 166-173 and new Supplemental Figure S6 and S7.

See Comment 3-9 below for more details.

**5. Figure 2 e and f evaluated how sequencing depths affected allelic calling, which is an important confounding factor. In this case, it would informative also to compare scBASE versus unique-reads methods.**

**Comment 2-5:** We agree and have added these comparisons to new Figure 3. These new comparisons confirmed that combining information improves estimation of allele proportions in low expressed genes.

**6. The idea of partial pooling can overcome the problem of scare read counts. But one allelic bursting is often random and dynamic. When pooling, it was shown that allelic calling canceled out each other. It is not clear to me how the pooling will help for random allelic expression. Pooling will be only helpful if the gene is imprinted, which means that the allelic expression is consistent across all the cells.**

**Comment 2-6:** In scBASE, pooling happens among cells in the same ASE states for each gene. The three-component mixture model in scBASE enables the classification of cells with respect to each cell's ASE state. Therefore, pooling does not average the allele proportions across ASE classes. We have added text to clarify this point in Discussion (Line 153-160).

**7. I am not a mathematician. But I wonder how to benchmark the validity of weighted allocation to make sure that it doesn't introduce false positive as well since it is also based on possibility score. What is the parameter setting that can affect allelic calling? It would be great to discuss this.**

**Comment 2-7:** The weighted allocation approach is not new – RSEM (Li et al. 2011 BMC Bioinformatics), AlleleSeq (Rozowsky et al. 2011 Mol Syst Biol.), kallisto (Bray et al. 2016 Nature Biotech.). Weighted allocation using expectation maximization algorithm does not generally assign reads to alleles without any reads uniquely aligning to it. However, allele-specific read counts after disregarding the reads that align to both alleles can result in increased rates of monoallelic (false negative). scBASE model parameters are estimated from the data using weakly informative prior distributions so there are no pre-set parameters that affect estimation of allele proportions.

**8. As author also mentioned (ref 15) several allelic methods are available, such as scphaser (scphaser: haplotype inference using single-cell RNA-seq data), which takes advantage of the allelic calling nearby the reads. It is extremely important to benchmark scBASE with the published methods. Also, the data scale used is still very small. Recently, many data points are available with hybrid single-cell sequencing (e.g. Chen et.al Genome Research, PMID 27486082).**

**Comment 2-8:** The scphaser software (Edsgärd 2016 Bioinformatics) exploits frequent monoallelic expression in single cells and reconstructs haplotypes by co-clustering major and minor SNVs. The major goal of scBASE is to improve the estimation of allelic proportion by combining the information across the cell population. The goal of each method is distinct and therefore direct benchmarking is not possible. For the F1 hybrid mouse we recommend that reads be aligned to a phase-known diploid transcriptome – this is a best-case scenario for phasing. When dealing with more complex genomes, phasing should be performed beforehand if haplotype-specific transcriptomes are not available and scphaser is one possible approach. We have added new text to point to this suggestion (Line 194-197).

**Reviewer #3 (Remarks to the Author):**

**In this study, the authors present a method, scBASE, to estimate Allele Specific Expression of a gene from single cell transcriptome data. Their method is supposed to deal with low coverage issues and allele dropout. Based on the presented data I find it difficult to evaluate the impact of the proposed algorithm on sc ASE analysis. My main concern is that a significant part of the manuscript is based on the concept of multi-mapping reads. In the results they claim that « In a typical scRNA-seq workflow for ASE [..] only the unique 14.9% of the original sequence reads « are available for the analysis. And this result comes from the fact that 59.3% of reads have to be thrown away because «allelic multi-reads« i.e. not univocally mappable given the presence of (heterozygous) variances in the genome. It is quite difficult to comment on this statement. If that was true, dozens of papers on single cell transcriptomes should be retracted. But also the whole literature on genomic variations, eQTL and clinical genetic applications based on RNAseq and Whole Exome or Genome sequencing should be thrashed.**

**In reality, any aligner, including Bowtie, has no problem to assign a read to the proper allele (normally 90% of reads in RNAseq or scRNAseq experiments have perfect mapping quality and usable for the analysis) which makes all the first paragraph of the results meaningless. Are the authors capable to prove this statement wrong using BWA, Bowtie, Star and other aligners? Could they please show the distribution of mapping quality per read in the experiments they cite in the manuscript and prove that 85% of the reads are unusable for ASE analysis? What happen if all reads are used irrespective of their mapping quality? If they cannot prove that, they need to show that the statistical recovery of the remaining 10% of reads (even less since many of the unmapped reads are not representing a biological signal) gives a significant improvement for ASE estimation.**

**Comment 3-0-1:** While we would not go so far, the reviewers' concerns do indicate that the issues we raise here are worthy of attention. We agree that many aligners will be able to align most reads. In fact, bowtie aligned 76.0% of all the reads to the diploid transcriptome in the case of Deng et al. data. Among the aligned reads, 74.2% aligned uniquely to the genes. However, 79.9% of these gene unique reads cannot distinguish between the two allelic copies due to lack of polymorphisms. A larger proportion of aligned reads (88.6%) were discarded by Deng et al. (https://www.ncbi.nlm.nih.gov/geo/download/?acc=GSE45719&format=file) due to allelic mapping ambiguity. The perfect alignment of a large majority of reads to both alleles is a property of the genome, not of the alignment algorithm or the read quality. It seems at first natural to discard these reads when estimating ASE parameters but we show that estimation can be improved by retaining and resolving them.

**The authors seem to criticize the usage of threshold ("arbitrary") in many other study but the whole manuscript is based on arbitrary choices:**

**Comment 3-0-2:** We did not intend the term arbitrary to be interpreted as criticism. Of course, one has to draw lines somewhere. We rephrased it in Line 269-273.

**1) What is the rationale of using a beta-binomial and half-Cauchy distributions with arbitrary parameters (2,7) as reported in the supplementary methods? (consider another study PMC5339288 using beta-binomial for ASE)**

**Comment 3-1:** Half-cauchy is a distribution for the hyperparameters (parameters of the prior distribution in our hierarchical model) and the parameters we chose are often used in a typical Bayesian analysis to approximate a non-informative state. These choices are somewhat arbitrary but importantly, they can easily be changed in the scBASE MCMC implementation and one could evaluate the impact of these choices. There may be alternatives that give even better performance than we have presented, but it is more likely that differences will be negligible.

**2) From where the authors derived the thresholds 0.7 and 0.9 in the paragraph "Classification of a gene according to its ASE profile across many cells"**

**Comment 3-2:** These are arbitrary boundaries that are useful to support a descriptive interpretation of the data. The classification probabilities are exact and specify precise points in the simplex but we found that rough classifications were useful to give a sense of the extent of, for example, maternal monoallelic expression in a population of cells. We introduced these seven categories of ASE profile to broadly describe and compare ASE dynamics of a cell population. We have added new text to clarify this point (Line 283-285).

**3) From where the authors derived the thresholds 0.02 and 0.98 in the paragraph "calling monoallelic expression from no-pulling estimators of allelic proportion"**

**Comment 3-3:** This is a simple and common way to call monoallelic expression using allele proportions from read counts. Varying these cut points within a few percentage points up or down had little impact on our conclusions. This is how all previous analyses of monoallelic expression have been done. On the other hand, scBASE classifies cells into monoallelic and bi-allelic classes by fitting the hierarchical mixture model, and therefore, enables us to compute classification probabilities without setting arbitrary thresholds. We believe this probabilistic classification is one of the strengths of our approach.

**4) They claim that beta distribution parameters are gene specific. However, they did not explained how these parameters change with respect to genes features. Gene length? GC content? Etc..**

**Comment 3-4:** scBASE allows parameters to have different values for different genes. This makes sense since the shape of distributions for monoallelic and bi-allelic classes can be different among genes depending on the numbers of SNPs or indels that distinguish two alleles. There is an interesting idea here though. Gene length is related to density of SNPs and indels. GC content may alter the information content of a gene sequence and influence alignment specificity. If gene length or GC content is related to allelic expression, it should be possible to discover these relationships in the data but whether they are present or not, the model is already designed to accommodate this kind of variation.

**Downsampling the data to evaluate the performance of the method is a good idea.**

**Comment 3-5-0:** Thanks. We followed the example of others, e.g. Huang et al [17].

**5) The reference estimation should be performed with both weighted allocation and unique-reads (all of them) on the full dataset.**

**Comment 3-5:** We revised our simulation tests and compared unique-reads versus scBASE counts for ground truth based upon weighted allocation and again based on ground truth for unique reads. In short, scBASE does not depend on how its input counts are estimated. We are now able to show that the classification and partial pooling of scBASE improves estimation using unique read counts as input (new Figure 3). Thanks for the suggestion.

**6) Really the authors removed 85% of the reads in the unique-reads approach when comparing their method? please perform the comparison using 1) all the reads 2) different thresholds of mapping quality**

**Comment 3-6:** This is a key point – all previous analyses of ASE in single cells have actually used only the reads aligned to specific alleles, disregarding the majority of reads. Our algorithm retains them. However, one has to first resolve the mapping ambiguity. The more permissive the threshold of mapping quality, the more reads would be multi-mapped between alleles. In fact, a majority of reads have good mapping quality in our approach. But a large proportion of them align to those regions in which no variants exist, and therefore are ignored when estimating allelic proportions or allele-specific expression from uniquely mapping reads.

**7) Could they validate their method with known imprinted genes and X inactivated genes during embryonic differentiation?**

**Comment 3-7:** An excellent suggestion. We have provided examples of some X chromosome and imprinted genes in new Supplemental Figure S5.

**8) It is not clear when MCMC or EM to estimate pgk is used. What are the advantages of one approach over the other?**

**Comment 3-8:** MCMC is a sampling-based method that approximates a posterior distribution of allelic proportions and parameters. EM provides point estimation and is much faster but less flexible than MCMC. By flexible, we mean that the model assumptions and parameters are easy to change in the MCMC code. All results in the manuscript used the MCMC code, and we have clarified this point in Line 216-218.

**There are quite sparse statements about the dynamics of transcriptional bursting activity. 9) Could the authors make a specific paragraph and support with factual arguments statements such as "SCALE with scBASE [...] will results in more accurate estimates of bursting kinetics" or "We also found that these data do not support the independence of allelic transcriptional bursts". In which genes the alleles are not independently transcribed? Is logOR sufficient to make this statement? No, if these genes are independently but frequently transcribed. What is the correlation between estimated ASE and transcriptional level? Do their ASE correlate with half-life RNA?**

**Comment 3-9:** We agree that the statements were vague. We have now run the SCALE analysis to confirm our claim (see Comment 1-4, 2-4, and Supplemental Figure S6 and S7).

Determining the dynamic parameters of allelic bursting from static single cell data requires assumptions about the frequencies, intensities, the spans of bursting, and the kinetics of RNA turnover. These issues are addressed in the Reinius et al. (2015 Nature Reviews Genetics) paper. Here we focused on the two-way cross-classification of cells at the sampled time points. Assuming scRNA-Seq captures a steady state of bursting and degradation for the most of genes, logOR [14] is used to estimate how often we see bi-allelic, monoallelic or no expression of two alleles. According to the logOR distribution, scRNA-seq data does not support independent allelic bursting for most genes. This is also observed in the original analysis of SCALE (https://github.com/yuchaojiang/SCALE).

We agree that for frequently bursting genes it is difficult to evaluate independence, which is why we only tested genes with sufficient representation of cells in each allelic expression state. In the original analysis of SCALE (see Figure R2 below), the majority of dynamic genes (in the orange circle) still deviate from the "perfect independence" curve (blue), leaning towards perfect dependence line (green). Compare this with the right panel in which perfect independence is assumed given the marginal probability of allelic bursts. Thus, although the data points do roughly follow the shape of the perfect independence curve in (A) many points lie above the curve. For the simulated independence data in (B), points are entirely below the reference curve.

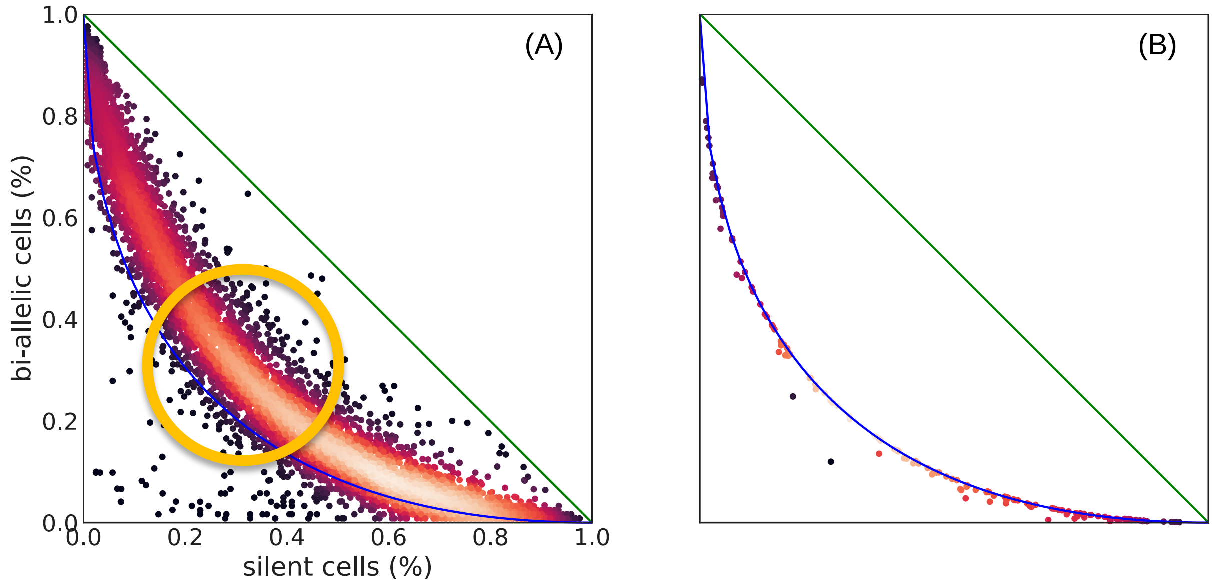


Figure R2: Proportion of silent and bi-allelic cells across genes using the original analysis of SCALE. Blue curve depicts the positions where perfect independence of allelic bursting is assumed, whereas green line is for perfect dependence. Most genes are located above the blue line, including dynamic genes (points in orange circle) (A). SCALE also estimates marginal allelic bursting probability, $P_M$ and $P_P$, and we computed the expected proportion of silent and bi-allelic cells assuming independence. When we do not assume $P_M = P_P$ as in Deng et al analysis, independence curve becomes a region (below the reference curve (B). Based upon this observation, we concluded that Deng et al. approach also supports non-independence of allelic bursting.

To better understand the relationship between Deng's method for evaluating independence and the odds ratio, it is helpful to consider the geometry of the 2x2 table probabilities (Figure R3). Each cell in a population can be in one of four states (not expressed, bi-allelic, maternal or paternal expression). The state probabilities must sum to one, so they fill out a 3D tetrahedral shape -- the 4D simplex [15]. The independence model corresponds to a region that satisfies the constraint $(P_B * P_N)/(P_M * P_P) = 1$ represented as the curved 2D region inside the simplex. Observed cell proportions under the independence model should cluster around this 2D region. If we impose the constraint that maternal and paternal gene expression are equally likely (as in Deng et al), this constrains the model to a planar cross-section of the simplex cutting across the line $P_M=P_P$ as indicated in the figure. The Deng model of perfect dependence falls on a line at the upper left edge of the simplex and the Deng model of perfect independence is a 1D curve where the two surfaces of constraints $(P_B * P_N)/(P_M * P_P) = 1$ and $P_M=P_P$ intersect. We note that the region of independence (i.e., where the odds ratio = 1), when projected onto the plane $P_M=P_P$ will fall entirely below the perfect independence curve, as seen in Figure R2b, and not "around" the curve as conjectured by Deng et al.

[redacted]

[redacted]

Reviewers' comments:

Reviewer #1 (Remarks to the Author):

My previous concerns are appropriately addressed. I don't have any additional comments.

Reviewer #2 (Remarks to the Author):

I appreciate that the authors addressed all the questions and also validated scBASE with SCALE. The concern remained to me is still the claim about the synchronization of allelic bursting. The authors should be aware that 286 preimplantation cells are special in the way that they represent the developmental progression coupling with zygotic genome activation and cell fate specification. The authors treated cells as a whole but not examined them for each developmental stage to investigate allelic burst synchronization, which is different from what Deng et.al have done. The authors need to check their claim at each specific developmental stage. Also, it still would be great that they can validate this claim in another bigger allelic dataset (e.g. Genome Research Chen et.al 2016, Nature Genetics Reinius et.al 2017, Nature Larsson et.al 2019). This is because more and more evidence is clearly pointing to independent allelic transcription.

Reviewer #3 (Remarks to the Author):

The authors made some effort to clarify the points I raised during the first review.
Despite some interesting ideas I still find the study incomplete and the manuscript not clear/not precise.

1) the proposed method, to be used, requires phased genotypes which are in general not available. Phasing with scphaser is proposed but not performances are not tested.
2) it is still not clear in the manuscript which samples have been used and when to test the algorithm. They say "we restricted attention to 13,006 genes with at least 4 allelic unique reads in at least
37 10% of cells (32 out of 286 cells)"
- 32 cells are not sufficient to draw any conclusion. there are (unphased) datasets with hundreds cells available (i.e. PMID: 29022598, PMID: 27668657, PMID: 30510006). Please use them

But then they say " The data provided by Deng et al. have relatively high coverage of ~13.8M reads per cell across the 60 cells from the mid-blastocyst stage".
In figure legends it seems they used 286 cells. so 32,60 or 286?
In any case the sample size is quite small for some of the conclusions drawn in the paper (see below).

3) I still have problems with the definition of multi-allelic reads in the manuscript. Are they uninformative reads with respect to ASE? To be clear, not overlapping with any "heterozygous sites"? Assuming this is the case then the example in Figure S1 and S5 are inconsistent. According to the sentence " Weighted allocation does not generally
assign reads to an allele when there are no reads uniquely aligned to it.", the gene Cdk2ap1 should have no reads after weighted allocation on C57BL/6J. How does the algorithm really work?

4) The authors failed to propose a convincing control for imprinted and X inactivated genes. They analysed only one gene Impact. scBASE and unique reads method yielded the same result (i.e. the plot is 99% the same with apparently less mistakes in the unique reads method).

Same for XCI. XIST is expected to be strongly monoallelically expressed (100% reads from the inactive chromosome). According to the reported numbers, scBASE gives more false positive calls 2% vs 1% from Unique reads.
The authors need to provide more evidence on imprinting and x-inactivation that scBASE is providing an advantage to currently used procedures in presence and in absence of phased genotypes.

5) The authors failed to cite the current literature on sc ASE . They ignored most of recent single cell ASE studies published so far (based on unique reads count) and only refer to a 2015 study (Deng et al): i.e. PMID: 29022598,PMID: 30510006, PMID: 28190458 among others.
They cite Reinius et al. (PMID: 27668657) but they do not make use of the (phased) dataset.

6) the small size of the dataset and the suspect of potential inflation of false positive calls invalid all paragraph about allelic bursting independence. I want to stress to the author that this is an important point, potentially leading to the investigation of new molecular mechanisms and deserve a much deeper analysis with multiple validations.

Minor:
1) comparisons MSE. How Figure 3 has been done? To me a huge positive difference MSE(scBASE) - MSE(unique reads) means that scBASe is wrong. Can they clarify?
2) They say that "All results in the manuscript used the MCMC code". So why talk about a never used EM code? It is confusing
3) They tested three methods but in the discussion they report ". We applied [...] each of four estimation methods to the down-sampled data"

# Reviewers' comments:

## Reviewer #1 (Remarks to the Author):

**My previous concerns are appropriately addressed. I don't have any additional comments.**

**Comment 1:** Thank you.

## Reviewer #2 (Remarks to the Author):

**I appreciate that the authors addressed all the questions and also validated scBASE with SCALE. The concern remained to me is still the claim about the synchronization of allelic bursting. The authors should be aware that 286 preimplantation cells are special in the way that they represent the developmental progression coupling with zygotic genome activation and cell fate specification. The authors treated cells as a whole but not examined them for each developmental stage to investigate allelic burst synchronization, which is different from what Deng et.al have done. The authors need to check their claim at each specific developmental stage. Also, it still would be great that they can validate this claim in another bigger allelic dataset (e.g. Genome Research Chen et.al 2016, Nature Genetics Reinius et.al 2017, Nature Larsson et.al 2019). This is because more and more evidence is clearly pointing to independent allelic transcription.**

**Comment 2-1:** It seems that we had failed to make it clear that we had applied the independence analysis to only the 122 mature blastocyst cells as in Jiang et al. We have made some modification to the main text (**Line 77-80**). We added a new section in Methods (**Line 383-402**) to explain how scBASE can be applied to subpopulations of cells within a single experiment.

We have now extended our application of scBASE to include the three data sets suggested by the reviewer. Each of these analyses reinforces our conclusions about the statistical independence of bursting. In order to incorporate these findings in the revised manuscript we have included additional supplemental material (Supplemental Figures S3, S4, and S5).

It was not our intention in the original submission to focus on the question of evaluating the independence of allelic bursting. However, based on the reviewers' comments and our further analysis of the problem, we feel that is a question of importance that scBASE software can help to address. Therefore, we have added a new paragraph to the Results (**Line 99-140**) and a new figure (Figure 4 and Supplemental Figures S2, S3, S4, and S5) to focus on the evaluation of independence. We also added a Discussion paragraph that addresses our interpretation of independence (**Line 261-280**). The reviewers' concern may reflect a distinction between the

statistical definition of independence and a biological concept of independent regulation of allelic expression. Statistical independence, if it holds, has profound implications for any setting in which it can be demonstrated. Specifically, there can be no causal connection, direct or indirect, between the variables (See Shipley, Cause and Correlation in Biology, first edition, section 2.13). Thus, statistical independence would preclude any global regulation of gene expression. From a biological perspective, independent regulation could be interpreted as the presence of any locally autonomous mechanism that is not coordinated across alleles. We believe, and the data support, a model in which gene expression regulation has both global and local components. We thank the reviewers for challenging our thinking on this topic and we hope they find our interpretation to be reasonable and satisfactory.

## Reviewer #3 (Remarks to the Author):

**The authors made some effort to clarify the points I raised during the first review. Despite some interesting ideas I still find the study incomplete and the manuscript not clear/not precise.**

**1) the proposed method, to be used, requires phased genotypes which are in general not available. Phasing with scphaser is proposed but not performances are not tested.**

**Comment 3-1:** Phasing is assumed in most algorithms [14, 15] for estimating allele-specific expression. For model organisms like mouse, parental genomes often are available and phasing is trivial. In other contexts, including human cells, most phasing software will provide a reliable local solution. Phasing methods are reviewed in-depth, for example, in Browning & Browning (2011). If phasing is impossible, ASE can be estimated at each SNP to which scBASE algorithm is still applicable. Evaluation of phasing methods falls outside the scope of study.

**2) it is still not clear in the manuscript which samples have been used and when to test the algorithm. They say "we restricted attention to 13,006 genes with at least 4 allelic unique reads in at least ~10% of cells (32 out of 286 cells)" - 32 cells are not sufficient to draw any conclusion. there are (unphased) datasets with hundreds cells available (i.e. PMID: 29022598, PMID: 27668657, PMID: 30510006). Please use them**

**Comment 3-2-1:** The quoted statement describes how we filtered the genes, not cells. In order to have reliable allelic expression estimates, we wanted to ensure that allelic unique reads were present in at least some of the cells. There are no analyses in the manuscript that apply to 32 cells.

**But then they say " The data provided by Deng et al. have relatively high coverage of ~13.8M reads per cell across the 60 cells from the mid-blastocyst stage". In figure legends it seems they used 286 cells. so 32,60 or 286? In any case the sample size is quite small for some of the conclusions drawn in the paper (see below).**

**Comment 3-2-2:** We used different numbers of cells in different analysis. We have made additions to the text to indicate where (and why) different sets of cells were used. The Deng et al. data is composed of heterogeneous cell types along the time course of mouse embryo development. Reviewer 2 had also raised the point that independence of allelic bursting could be confounded by the highly biased ASE in early developmental cells (zygotes, 2-cells, etc). Therefore, we used only the 122 mature blastocyst cells in the analysis of independence (Figure 4). In order to minimize the use different subsets of data, we repeated the evaluation of scBASE in Figure 3 using the same set of 122 cells, where previously we had used 60 cells from the mid blastocyst state. In our analysis of the impact of numbers of cells on the standard error of estimation (requested in the last round of reviews), we compare the SEs obtained with 286 cells to SEs from 60 cells (Supplemental Figure S8) to provide a robust illustration of the impact of reducing the number of cells. All other analyses of the Deng et al. data use the full set of 286 cells. This includes the analysis in Figure 5 where we break down ASE according to cell type-specific subpopulations. The classification of ASE within subpopulations is explained with new text in Methods (Estimating allelic proportions in subpopulations of cells). We now indicate in both the text and figure captions the number and types of cells used in each analysis. We hope that this will reduce any potential for confusion.

| **Assessment type** | **Cell Types** | **Number of cells** | **Note** |
|---|---|---|---|
| Effect of maintaining multi-reads (**Figure 1**) | Pre-implantation mouse embryos from zygotes to blastocysts | 286 | We want to assess how the number of monoallelic genes changes along the time course of embryo development. (See **Line 62-64**) |
| Effect of partial pooling (**Figure 2**) | Mature blastocysts | 122 | scBASE should be applied to a homogeneous group of cells. The largest number of cells are from mature blastocyst stage in Deng et al. (See **Line 78-80**) |
| Independence of allelic bursting (**Figure 3**) | Mature blastocysts | 122 | Jiang et al. performed their analysis including independence test on these cells. (See **Line 120-121**) |
| Effect of cell counts on model fitting (**Supp. Figure S8**) | Mid-blastocysts | 60 | According to Reviewer 1's suggestion, we assessed how standard deviation on allelic proportions changes between using 286 cells and 60 cells. (See **Line 234-237**) |
| Cellular proportions of ASE states (**Figure 5**) | Pre-implantation mouse embryos from zygotes to blastocysts | 286 | We want to assess how the proportion of cells in different allelic expression states changes along the time course of embryo development. (See **Line 170-172**) |

**3) I still have problems with the definition of multi-allelic reads in the manuscript. Are they uninformative reads with respect to ASE? To be clear, not overlapping with any "heterozygous sites"? Assuming this is the case then the example in Figure S1 and S5 are inconsistent. According to the sentence " Weighted allocation does not generally assign reads to an allele when there are no reads uniquely aligned to it.", the gene Cdk2ap1 should have no reads after weighted allocation on C57BL/6J. How does the algorithm really work?**

**Comment 3-3:** See new text that address the definition of multi-reads (**Line 57-62**). Of all reads in the Deng et al. data, 23.3% are complex multi-reads. The critical reads for the example in Figure S1 (highlighted in gold) are genomic multi-reads that are allelic-unique. These reads convey information about allelic expression and, because *Cdk2ap1*, is expressed at a substantially higher level compared to the other genes, the EM algorithm estimates a non-zero expected read count for the B6 allele of *Cdk2ap1*.

**4) The authors failed to propose a convincing control for imprinted and X inactivated genes. They analysed only one gene Impact. scBASE and unique reads method yielded the same result (i.e. the plot is 99% the same with apparently less mistakes in the unique reads method).**

**Comment 3-4-1**: In this revision, we report additional results from our analysis of putative imprinted genes and X chromosome genes across 3 different sets of data (Deng et al. [7], Reinius et al. [23], and Chen et al. [24]).

In Supplemental Figures S12 and S13, we analyzed a set of 158 putative imprinted genes from [25, 26, 27]. It appears that in the cell types represented by these 3 experiments, imprinting is not manifested for most of these genes. This observation holds across all methods of estimation including the unique-reads method. Several genes turn out to contain more bi-allelic cells if we disambiguate multi-reads or if we perform partial pooling, for the reasons we discussed in Supplemental Figure S1. However, there are several clear examples of imprinting too. For example, in the Reinius et al. reported 15 genes within the 158 putative genes. Using scBASE we identified 14 genes (B830012L14Rik, Gpr1, **Igf2r**, **Impact**, Mecp2, **Meg3**, **Mirg**, Ndn, **Peg3**, Peg10, **Peg12**, **Plagl1**, Pon3, **Rian**) after weighted allocation and partial pooling – 8 of them (in bold text) are common to what Reinius et al. [23] identified.

**Same for XCI. XIST is expected to be strongly monoallelically expressed (100% reads from the inactive chromosome). According to the reported numbers, scBASE gives more false positive calls 2% vs 1% from Unique reads. The authors need to provide more evidence on imprinting and x-inactivation that scBASE is providing an advantage to currently used procedures in presence and in absence of phased genotypes.**

**Comment 3-4-2:** We applied scBASE to estimate allelic expression in female X chromosome genes from 3 data sets [7, 23, 24]. We estimated the allelic proportion of Xist gene expression and the ASE state of all X chromosome genes from female cells using (i) unique reads, (ii) weighted allocation, (iii) unique reads with partial pooling, and (iv) weighted allocation with partial pooling in Deng et al., Reinius et al., and Chen et al., data sets. Xist was largely monoallelic in mouse primary fibroblast cells [23] (Supplemental Figure S9a). Partial pooling corrects allelic proportion of Xist towards either of maternal or paternal monoallelic expression in many cells for both (iii) unique reads and (iv) weighted allocation. XCI was consistent with the allele specificity of Xist (Supplemental Figure S9b). Partial pooling also corrects the profiling of ASE states for both unique reads and weighted allocation, and strengthens the ASE pattern conforming to the XCI onset. On the other hand, Xist expression was clearly bi-allelic in many mESCs, mEpiSCs, motor neuron cells [24] (Supplemental Figure S10a), and pre-implantation embryo cells [7] (Supplemental Figure S11a) irrespective of estimation method used. This suggests that XCI is incomplete in these cells and this is reflected in the varied allelic expression patterns of X chromosome genes in these cells (Supplemental Figures S10b and S11b).

**5) The authors failed to cite the current literature on sc ASE . They ignored most of recent single cell ASE studies published so far (based on unique reads count) and only refer to a 2015 study (Deng et al): i.e. PMID: 29022598,PMID: 30510006, PMID: 28190458 among others. They cite Reinius et al. (PMID: 27668657) but they do not make use of the (phased) dataset.**

**Comment 3-5:** We have updated our references, some of which appeared subsequent to the first submission of our manuscript. And we have applied scBASE to data from three additional, recent publications (Reinius et al. [23], Chen et al. [24], and Larsson et al. [20]).

**6) the small size of the dataset and the suspect of potential inflation of false positive calls invalid all paragraph about allelic bursting independence. I want to stress to the author that this is an important point, potentially leading to the investigation of new molecular mechanisms and deserve a much deeper analysis with multiple validations.**

**Comment 3-6:** Please see our response to reviewer 2 (Comment 2-1) with regard to the independence of bursting.

**Minor:**

**1) comparisons MSE. How Figure 3 has been done? To me a huge positive difference MSE(scBASE) - MSE(unique reads) means that scBASe is wrong. Can they clarify?**

**Comment 3-7:** The y-axis of those plots are the difference in MSE, MSE(unique reads) - MSE(scBASE). We have now clarified this in the main text and in the figure caption.

**2) They say that "All results in the manuscript used the MCMC code". So why talk about a never used EM code? It is confusing**

**Comment 3-8:** We provide clarification in the in the new Methods section (Estimating allelic proportions in subpopulations of cells, **Line 383-402**) and in the results (**Line 169-176**). The problem that we encountered is that the EM algorithm can be unstable when estimating the parameters of the monoallelic expression states. We recommend two options. The first one is to run MCMC implementation of scBASE for each cell type. The strength of this approach is that it provides the posterior distribution of all the parameters. But the level of uncertainty increases for estimated parameters when the number of cells in any cell type is limited. The second option is to run MCMC with all the available cells and estimate the prior (shape and scale) parameters. These parameters describe how allelic proportions of cells in each bursting state (monoallelic or bi-allelic) distribute, and therefore, common across cell types. This strategy stabilizes the parameter estimation as more cells are used. Then we can fix the estimated prior parameters and re-estimate model parameters for each cell type. For this purpose, we found that the Expectation-Maximization (EM) algorithm is helpful because it executes quickly and converges to the maximum a posteriori parameter estimates. In the revised manuscript we have applied the second strategy. Changes in the results (Figure 5) are negligible and the computation was substantially faster.

**3) They tested three methods but in the discussion they report ". We applied [...] each of four estimation methods to the down-sampled data"**

**Comment 3-9:** Based on this comment, we agree that the different options available in scBASE were not clearly laid out. Following the reviewers' suggestion in the previous revision, we now provide ASE estimation using unique reads with partial pooling. Therefore, there are four different methods that we can apply to estimate allelic proportions: (i) unique read counts, (ii) weighted allocation counts, (iii) unique read counts with partial pooling, and (iv) weighted allocation counts with partial pooling. We have now taken care to spell out these options early in the manuscript (**Line 31-36**). And wherever it is reasonable we have applied and evaluated all four methods (e.g. Figure 4c and Supplemental Figures S2, S3, S4, S9, S10, S11, S12, and S13). In particular, we modified our benchmarking tests (Figure 3) to evaluate each of these four estimation methods against two different truth standards. The first truth standard is the unique-reads estimate based on the full data and the second truth standard is the weighted allocation estimate based on the full data. We trust that this provides a thorough set of evaluations and uses truth standards that have different potential biases.

Reviewers' comments:

Reviewer #2 (Remarks to the Author):

I appreciate that the authors have made a significant improvement to their manuscript by analyzing suggested datasets. In general, each algorithm has its own weakness and can never be perfect. It is a good initiative to use a statistical approach and make use of multi-mapped reads. I am positive with the revision but still have some remaining comments:

1. Line 14-16, sentences in red fit better the context if put before “Allele-specific expression (ASE) in single cells can provide a rich picture of the stochastic and dynamic properties of gene expression in individual cells ”.

2. Line 58-60, the authors failed to explain what are complex multi-reads

3. “We estimated unique reads and weighted allocation counts from each individual cell using all 286 cells to show how the number of monoallelic genes changes along the developmental time course”. Here should indicate which figure to refer to.

4. Figure 2a, it is interesting to know what are those genes that fall out of monoallelic genes if use weighed allocation in zygotes and 2-cells? Also, ZGA is not initiated before 2-cell stage, I would expect that zygote should be a good control showing a similar level of ASE of unique and multi-mapped reads.

5. Line78, the authors claimed that they applied partial pooling to a homogeneous group of cells, hence 122 mature blastocyte cells. Maybe the authors are not familiar with the preimplantation development, during which blastocyte cells are least homogeneous since they start lineage specification, hence showing the reduced correlation in Deng et.al publication. I am skeptical about using these cells for pooling analysis.

6. Figure 3, a,b should have a subtitle in the plot for the easiness to read. The same for Figure 4.

7. In the discussion, the authors mentioned about imprinting genes are mostly showed allelic biased instead of only monoallelic expressed, which could be expected due to the sensitivity of single-cell sequencing. Can the authors summarize a table of such imprinting genes with a ratio of allelic expression in order for readers to follow up?

8. The authors used four methods, i.e. (i) unique reads, (ii) weighted allocation, (iii) unique reads with partial pooling, and (iv) weighted allocation with partial pooling implemented in scBASE. It should be discussed the performance of these methods, i.e. which one the authors recommend to use.

9. Line 227, In discussion: “we also observe that XCI is not fully established for these cells”. I would be careful to make such comments as Xist expression often suffers from technical issues with single-cell sequencing due to its long sequence. And Xist expression is not the only index for XCI evaluation.

Reviewer #3 (Remarks to the Author):

The authors have improved the quality of the manuscript clarifying most of the points raised by the reviewers.

However I still do not see a clear advantage of using weighted allocations versus the more simple and straightforward unique read count. The example they report is quite anedoctical and almost cherry-picking (579 reads mapping to one parental genome C57BL/6J, but in two genes so to be discarded by the unique read selection - quite a unique case I guess) and as it is cannot support their conclusions without any other evidence. More specifically:
1) How many genes they found with this condition? They must show for a substantial set of genes why they appear monoallelically expressed and how they can be recovered with scBASE as they did with Cdk2ap1.
Concerning the transcriptional bursting, I appreciate the way they interpret their results now. However they have to discuss why their results are substantially different from Deng et al. where only genes with RPKM>90 have been used.
2) Could the authors repeat the analysis using more RPKM thresholds?

Reviewers' comments:

**Reviewer #2 (Remarks to the Author):**

I appreciate that the authors have made a significant improvement to their manuscript by analyzing suggested datasets. In general, each algorithm has its own weakness and can never be perfect. It is a good initiative to use a statistical approach and make use of multi-mapped reads. I am positive with the revision but still have some remaining comments:

1. Line 14-16, sentences in red fit better the context if put before “Allele-specific expression (ASE) in single cells can provide a rich picture of the stochastic and dynamic properties of gene expression in individual cells ”.

**Comment 2-1:** We have edited the text as proposed (Line 12-16).

2. Line 58-60, the authors failed to explain what are complex multi-reads

**Comment 2-2:** We clarified the definition (Line 60-62).

3. “We estimated unique reads and weighted allocation counts from each individual cell using all 286 cells to show how the number of monoallelic genes changes along the developmental time course”. Here should indicate which figure to refer to.

**Comment 2-3:** We specified the figure in which the comparison was made (Line 65-66).

4. Figure 2a, it is interesting to know what are those genes that fall out of monoallelic genes if use weighed allocation in zygotes and 2-cells? Also, ZGA is not initiated before 2-cell stage, I would expect that zygote should be a good control showing a similar level of ASE of unique and multi-mapped reads.

**Comment 2-4:** We found both unique reads and weighted allocation identified biallelic genes, although we get more biallelic genes using weighted allocation. For example, in zygote cells, the unique-reads method and weighted allocation identified 1,622 and 3,317 biallelic genes, respectively. The original Deng et al. analysis (downloaded from https://www.ncbi.nlm.nih.gov/geo/query/acc.cgi?acc=GSE45719) also report 884 biallelic genes. This suggests that hundreds or thousands of genes are already expressed from the paternal genome in these early embryo cells irrespective of which read count estimation strategies we use.

We performed a functional enrichment analysis for those biallelic genes identified in zygote cells using both unique reads and weighted allocation (See Table R1 and R2). Many of these biallelic genes are related to initiation (or minor activation [Higuchi 2018 J. Reproduction and Development]) of zygotic genome activation, i.e., degradation of maternal transcripts, DNA replication, chromatin remodeling, epigenetic modification, TF activities, and cell cycle. More genes (~79%) were identified by weighted allocation in each common term of molecular functions compared to unique-reads method. Most extra biallelic genes identified by the weighted allocation method were also related to these common molecular function terms, enhancing the characterization of zygotic genome activation. We concluded that weighted allocation may provide a more accurate perspective on allele-specific expression on early-stage embryos.

Table R1: Functional enrichment of biallelic genes in zygote identified by both unique-reads and weighted allocation method

| **Molecular Functions** | **Unique reads** | **Weighted Allocation** |
|---|---|---|
| ubiquitin-like protein ligase binding | 50 | 97 |
| proximal promoter sequence-specific DNA binding | 61 | 116 |
| chromatin binding | 83 | 172 |
| promoter-specific chromatin binding | 13 | 21 |
| histone binding | 46 | 81 |
| methylated histone binding | 16 | 27 |
| methylation-dependent protein binding | 16 | 27 |
| modification-dependent protein binding | 28 | 51 |
| mRNA binding | 42 | 87 |
| mRNA 3'-UTR binding | 18 | 29 |
| double-stranded RNA binding | 22 | 31 |
| Rho GTPase binding | 37 | 54 |
| Ras GTPase binding | 64 | 108 |
| phosphatase binding | 33 | 58 |
| small GTPase binding | 64 | 111 |
| GTPase binding | 73 | 133 |
| tubulin binding | 50 | 85 |
| microtubule binding | 40 | 66 |
| ATP-dependent microtubule motor activity, plus-end-directed | 8 | 10 |
| **Total** | **764** | **1364** |

Table R2: Functional enrichment of biallelic genes in zygote identified by weighted allocation method alone

| Molecular Function | Weighted Allocation |
|---|---|
| ubiquitin-like protein transferase activity | 127 |
| ubiquitin-protein transferase activity | 123 |
| ubiquitin protein ligase binding | 94 |
| ubiquitin-like protein ligase activity | 69 |
| ubiquitin protein ligase activity | 67 |
| transcription coregulator activity | 128 |
| transcription coactivator activity | 76 |
| transcription regulatory region sequence-specific DNA binding | 166 |
| RNA polymerase II regulatory region DNA binding | 158 |
| RNA polymerase II proximal promoter sequence-specific DNA binding | 112 |
| RNA polymerase II regulatory region sequence-specific DNA binding | 155 |
| RNA polymerase II core promoter sequence-specific DNA binding | 10 |
| telomeric DNA binding | 17 |
| core promoter sequence-specific DNA binding | 15 |
| histone acetyltransferase activity | 27 |
| histone-lysine N-methyltransferase activity | 19 |
| histone deacetylase binding | 38 |
| nuclear hormone receptor binding | 50 |
| hormone receptor binding | 53 |
| thyroid hormone receptor binding | 12 |
| peptide-lysine-N-acetyltransferase activity | 28 |
| peptide N-acetyltransferase activity | 31 |
| N-acetyltransferase activity | 34 |
| N-acyltransferase activity | 37 |
| single-stranded RNA binding | 32 |
| pre-mRNA binding | 15 |
| translation regulator activity, nucleic acid binding | 11 |
| translation regulator activity | 21 |
| poly(A) binding | 12 |
| protein C-terminus binding | 69 |
| phosphoprotein binding | 30 |

5. Line78, the authors claimed that they applied partial pooling to a homogeneous group of cells, hence 122 mature blastocyte cells. Maybe the authors are not familiar with the preimplantation development, during which blastocyte cells are least homogeneous since they start lineage specification, hence showing the reduced correlation in Deng et.al publication. I am skeptical about using these cells for pooling analysis.

**Comment 2-5:** Our original description was not clear on this point. We clarified it in the manuscript (Line 80). For the partial pooling to be effective, **scBASE does not require *a cell type* to be composed of homogeneous cells** since the information pooling is among cells in the same ASE state. What we are trying to avoid is mixing cell types

where the allelic proportions are highly skewed, e.g., the early cell stages, compared to other later-stage cells. According to the suggestion in previous review, we followed Jiang et al. who also analyzed those 122 mature blastocysts for their bursting dynamics analysis.

6. Figure 3, a,b should have a subtitle in the plot for the easiness to read. The same for Figure 4.

**Comment 2-6:** We added annotations and captions to the figures. Thanks.

7. In the discussion, the authors mentioned about imprinting genes are mostly showed allelic biased instead of only monoallelic expressed, which could be expected due to the sensitivity of single-cell sequencing. Can the authors summarize a table of such imprinting genes with a ratio of allelic expression in order for readers to follow up?

**Comment 2-7:** We posted our estimates of allele-specific counts for all the genes across all the cells (ftp://churchill-lab.jax.org/analysis/scBASE/) so anyone can look up his/her genes of interest. These result files include imprinting genes as well as sex chromosome genes. We also share all the analysis scripts in the same online repository. However we keep drifting further from the original aims of the paper. We are not comfortable proposing new interpretation of experiments that we did not carry out. The exercise of looking at imprinted genes was helpful for illustrating the algorithm and we are simply reporting what we observe.

8. The authors used four methods, i.e. (i) unique reads, (ii) weighted allocation, (iii) unique reads with partial pooling, and (iv) weighted allocation with partial pooling implemented in scBASE. It should be discussed the performance of these methods, i.e. which one the authors recommend to use.

**Comment 2-8:** We wish to make all options available for people to explore and better understand the implications of each algorithm, since this is a method paper. We make a recommendation to use multi-reads and partial pooling – now restated at end of discussion (Line 287-291). But the best outcome for us is that people examine these methods in other contexts with their own data. This is the best way to improve understanding.

9. Line 227, In discussion: “we also observe that XCI is not fully established for these cells”. I would be careful to make such comments as Xist expression often suffers from technical issues with single-cell sequencing due to its long sequence. And Xist expression is not the only index for XCI evaluation.

**Comment 2-9:** We made the conclusion based on our observation of a clear difference between female fibroblast cells (Reinius et al.) and female embryo (Deng et al.) or epistem cells (Chen et al.). In fibroblasts, the expression of X chromosome genes were enriched in the opposite allele to which allele Xist is expressed from (Supplemental Figure S9). The embryo or epistem cells did not show evidence of established XCI (Supplemental Figure S10 and S11). We don't want to make biological conclusions about XCI, only to report what we see in our analysis in order to illustrate how the algorithm behaves (Also see Comment 2-7).

**Reviewer #3 (Remarks to the Author):**

The authors have improved the quality of the manuscript clarifying most of the points raised by the reviewers.
However I still do not see a clear advantage of using weighted allocations versus the more simple and straightforward unique read count. The example they report is quite anedoctical and almost cherry-picking (579 reads mapping to one parental genome C57BL/6J, but in two genes so to be discarded by the unique read selection - quite a unique case I guess) and as it is cannot support their conclusions without any other evidence.

More specifically:

1) How many genes they found with this condition? They must show for a substantial set of genes why they appear monoallelically expressed and how they can be recovered with scBASE as they did with Cdk2ap1.

**Comment 3-1:** We found 2.5% of reads are simple genomic multi-reads (Line 66). Although they map to multiple genes, they are specific to one allele on each gene they align to. This amounts to on average over 410,000 reads per cell in Deng et al. embryo data. These reads contribute to the difference in the estimated number of monoallelic and biallelic genes in each cell (Figure 2).

Concerning the transcriptional bursting, I appreciate the way they interpret their results now. However they have to discuss why their results are substantially different from Deng et al. where only genes with RPKM>90 have been used.

2) Could the authors repeat the analysis using more RPKM thresholds?

**Comment 3-2:** RPKM is a way to normalize transcript lengths, and therefore, when considering only one gene at a time, as in independence testing, it is proportional to read counts. Setting the threshold up or down, did not change our conclusion. For consistency across four datasets, we have used the same count measure and threshold as in Larsson et al. in our independence evaluations. For example, Supplemental Figure S5a(i) is the same scatter plot as Figure 1(j) in Larsson et al. The difference is in how we interpret the plot of biallelic versus silent cell proportions. The independence tests proposed by us (and also by Jiang et al.) are the standard method widely used in most dependency tests. Deng et al. or Larsson et al. made an unnecessary assumption — the marginal bursting probability of maternal and paternal alleles are equivalent — and their interpretation of the plot is qualitative.

REVIEWERS' COMMENTS:

Reviewer #3 (Remarks to the Author):

The authors provided reasonably answers to most of my observation and I have only one last request. Concerning the analysis of bursting independence, given their results, the authors have to comment on the presence of potential known eQTLs driving allelic expression in these specific cells, and comment as in Jiang et al. that aseQTL (variants affecting allelic expression balance but not changing the overall gene expression) are not easily detectable in standard bulk eQTL studies.

In regard to the comment by reviewer 3:  It is an interesting point. There is abundant evidence to support the idea that allele-specific imbalance is (largely) driven by cis-acting genetic variants. These variants would be detectable as eQTL in genetic crosses or in population samples in single cell or bulk tissues. The reviewer proposes a scenario by which a genetic variant alters the allelic balance without altering the overall expression level of a gene. In order for this to happen, there would have to be some trans-acting feedback regulation of gene expression that would regulate both allelic copies. While it would be of interest, discussion would involve a substantial digression. As we have found no examples and have no novel insights to offer on this point, anything we add would be speculation. We appreciate the insights offered Reviewer 3 over the course of the review process (as well as those of the other reviewers) and as a result of the back-and-forth, we feel that the manuscript has broadened in its scope and potential impact.
